# Analysis of RNA decay factor mediated RNA stability contributions on RNA abundance

**DOI:** 10.1186/s12864-015-1358-y

**Published:** 2015-03-06

**Authors:** Sho Maekawa, Naoto Imamachi, Takuma Irie, Hidenori Tani, Kyoko Matsumoto, Rena Mizutani, Katsutoshi Imamura, Miho Kakeda, Tetsushi Yada, Sumio Sugano, Yutaka Suzuki, Nobuyoshi Akimitsu

**Affiliations:** Department of Medical Genome Sciences, Graduate School of Frontier Sciences, The University of Tokyo, 5-1-5 Kashiwanoha, Kashiwa, Chiba 277-8562 Japan; Radioisotope Center, The University of Tokyo, 2-11-16 Yayoi, Bunkyo-ku, Tokyo 113-0032 Japan; Research Institute for Environmental Management Technology, National Institute of Advanced Industrial Science and Technology (AIST), 16-1 Onogawa, Tsukuba, Ibaraki 305-8569 Japan; Department of Bioscience and Bioinformatics, Kyushu Institute of Technology, 680-4 Kawazu, Iizuka, Fukuoka 820-8502 Japan; Department of Computational Biology, Graduate School of Frontier Sciences, The University of Tokyo, 5-1-5 Kashiwanoha, Kashiwa, Chiba 277-8561 Japan

**Keywords:** BRIC-seq, ChIP-seq, Integrative analysis, Next-generation sequencing, RNA stability, Estimation of transcriptional level

## Abstract

**Background:**

Histone epigenome data determined by chromatin immunoprecipitation sequencing (ChIP-seq) is used in identifying transcript regions and estimating expression levels. However, this estimation does not always correlate with eventual RNA expression levels measured by RNA sequencing (RNA-seq). Part of the inconsistency may arise from the variance in RNA stability, where the transcripts that are more or less abundant than predicted RNA expression from histone epigenome data are inferred to be more or less stable. However, there is little systematic analysis to validate this assumption. Here, we used stability data of whole transcriptome measured by 5′-bromouridine immunoprecipitation chase sequencing (BRIC-seq), which enabled us to determine the half-lives of whole transcripts including lincRNAs, and we integrated BRIC-seq with ChIP-seq to achieve better estimation of the eventual transcript levels and to understand the importance of post-transcriptional regulation that determine the eventual transcript levels.

**Results:**

We identified discrepancies between the RNA abundance estimated by ChIP-seq and measured RNA expression from RNA-seq; for number of genes and estimated that the expression level of 865 genes was controlled at the level of RNA stability in HeLa cells. ENCODE data analysis supported the idea that RNA stability control aids to determine transcript levels in multiple cell types. We identified UPF1, EXOSC5 and STAU1, well-studied RNA degradation factors, as controlling factors for 8% of cases. Computational simulations reasonably explained the changes of eventual mRNA levels attributable to the changes in the rates of mRNA half-lives. In addition, we propose a feedback circuit that includes the regulated degradation of mRNAs encoding transcription factors to maintain the steady state level of RNA abundance. Intriguingly, these regulatory mechanisms were distinct between mRNAs and lincRNAs.

**Conclusions:**

Integrative analysis of ChIP-seq, RNA-seq and our BRIC-seq showed that transcriptional regulation and RNA degradation are independently regulated. In addition, RNA stability is an important determinant of eventual transcript levels. RNA binding proteins, such as UPF1, STAU1 and EXOSC5 may play active roles in such controls.

**Electronic supplementary material:**

The online version of this article (doi:10.1186/s12864-015-1358-y) contains supplementary material, which is available to authorized users.

## Background

The eventual RNA transcript level of a gene is determined by regulation at multiple levels, including transcriptional initiation, elongation, splicing, export and degradation. Transcription initiation is regulated by complex interactions of sequence features, many of which involve chromatin modifications [1]. Although it is still unclear whether chromatin modifications are the cause or consequence of transcription, these chromatin modifications are often used to infer transcriptional regulation. The chromatin modifications include several types of histone modifications, such as H3K4 tri-methylation (H3K4me3), which is often observed around the transcriptional start sites of actively transcribed transcripts [2,3]. In several large-scale projects they often used H3K4me3 sites as markers for active transcription, which allowed the characterization of transcriptionally active regions and estimation of transcript levels in a given cell at a given state [4]. This partly reflects the fact that advances in next generation sequencing have enabled easy characterization of the sites bound by H3K4me3 sites using chromatin immunoprecipitation sequencing (ChIP-seq) [5]. Indeed, a recent ENCODE study conducted a large number of ChIP-seq experiments in difference cell types. There have been several papers that modeled gene expression levels from chromatin features [4,6,7]. It is evident that ChIP-seq data is not sufficient enough to model the steady-state RNA expression levels for a number of genes, and regulatory mechanisms other than transcription initiation needs to be considered to understand the RNA expression.

RNA degradation is regulated by degradation factor, such as UPF1, EXOSC5 and STAU1, through RNA-protein and protein-protein interactions. UPF1 is an essential mediator in nonsense-mediated mRNA decay (NMD), in which aberrant RNA containing a premature stop-codon (PTC) is recognized and degraded [8-10]. In addition, recent genome-wide analyses by microarrays and RNA-seq have suggested a regulatory role for UPF1 in targeting 3-20% of *bona fide* mRNA with full coding potential [11-15]. UPF1 is involved in other degradation pathways such as the Staufen1-mediated mRNA decay (SMD) and replication-dependent histone mRNA decay [16,17]. It was proposed that approximately 1% of human mRNAs are regulated by Staufen1 (STAU1), suggesting that SMD constitutes a significant post-transcriptional regulatory pathway [18]. EXOSC5 is the essential component of the exosome complex, which functions in 3′ – 5′ RNA degradation [19,20]. However, even for these well-known factors, it is still unclear as to what extent they effect the eventual transcript levels.

In this study, we generated and integrated a dataset of BRIC-seq [21,22], RNA-seq and ChIP-seq, in order to uncover the contributions of RNA decay in determining eventual genome-wide transcript levels [23]. In BRIC-seq, the half-lives of transcripts are measured using 5′-bromouridine (BrU) based *in situ* labeling of RNA. BrU added in culture medium is incorporated into cells, which convert it to BrUTP. It is incorporated into nascent RNA during transcription, and consequently, endogenous RNAs are labeled with BrU. BrU-labeled total RNAs are isolated from cells at sequential time points after removal of surplus BrU from the culture medium. BrU-labeled RNAs are recovered by immunopurification followed by analysis by massive sequencing. By this method, we can avoid artificial effects of the traditionally used transcriptional inhibitor, such as actinomyicin D, method, in which the physiology of the cell is known to be greatly affected [22]. Although 5′-ethynyl uridine labeling and 4′-thiouridine labeling methods have been used for measuring the transcriptome stability, these nucleotide analogues are more toxic than BrU. BrU therefore has an advantage to determine RNA stabilities in physiologically non-disturbed conditions. It has been known that the RNA abundance does not necessarily correlate with their transcription rates; however the reasoning behind the lack of correlation, have not been well characterized. Here we identified genes that have low RNA abundance that could be explained by a particular RNA half-life. In addition, with the aid of computational simulation, we identified genes with RNA abundance that was mediated by changes in RNA stability by RNA decay factors: UPF1, EXOSC5, and STAU1.

## Results

### Correlation between ChIP-seq and RNA-seq data

First we analyzed the relationship between levels of the transcripts and the strength of active chromatin marks by performing ChIP-seq (chromatin immunoprecipitation) analysis of H3K4me3 and pol II on the Illumina HiSeq2000 platform. ChIP-seq peaks were called using a representative analytical program, MACS [24], using the default parameters (false discovery rate of p < 10^−5^). For the transcript levels, we used RNA-seq to determine the genome-wide gene expression in HeLa cells and we analyzed RNA-seq by modeling the gene to the Refseq gene model (for statistics, see Additional file 1: Figure S1).

We identified a total of 11,116 (2,732 with low peak and 8,384 with high peak) and 6,319 genes that possessed H3K4me3 and pol II “peak”, respectively, within the 3 kb regions around transcriptional start sites (+/−1.5 kbp, with transcriptional start sites designated as 0), out of 18,853 RefSeq genes analyzed (Table 1, Additional file 1: Figure S1a and Additional file 2: Table S1). In the cases where the peaks were not identified, the ChIP-seq tags were mostly at the noise level; if peaks were identified, we associated the signal intensities of ChIP-seq of H3K4me3 and RNA-seq gene expression levels (in RPKM) to the gene. We observed genes possessing more than 1 × 10^4^ H3K4me3 sequence tags and pol II peaks (“high peaks” group showed RNA level of >1 and >10 RPKM in 7,656 (73.5%) and 4,073 (84.0%) cases, among 10,421 and 3,069 genes with pol II peaks respectively (Table 1 and Additional file 2: Table S1).

**Table 1:** **Statistics of H3K4me3 ChIP-seq peaks against gene expression**

	**Total**	**No peak**	**Low Peak**	**High Peak**
**Number of Genes**	**18,853**	**7,737**	**2,732**	**8,384**
**Genes having RNA level of >1 RPKM**	**10,421**	**957**	**1,808**	**7,656**
**Genes having RNA level of >10 RPKM**	**4,848**	**175**	**600**	**4,073**

Peaks were called using MACS and “peak present” represents genes with H3K4me3 and pol II peaks within 1.5 kb of the TSS (details in Methods). “No peak” represents genes without any H3K4me3 or pol II peaks within 1.5 kb of the TSS.

**Figure 1:**
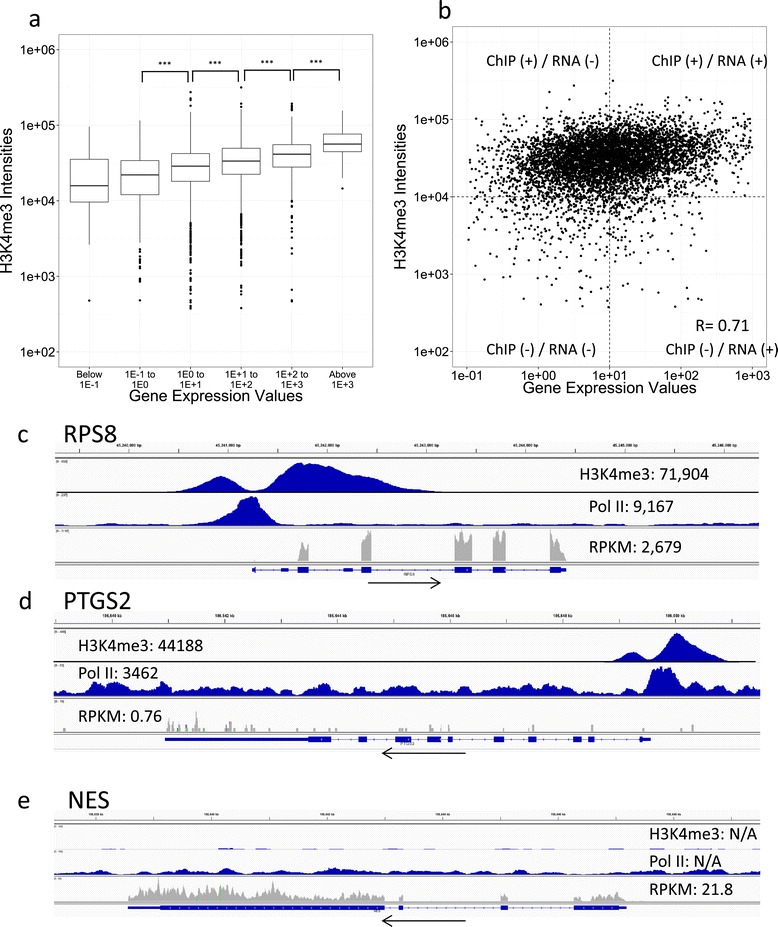
**Relationship between chromatin marks and eventual transcript levels. (a)** Boxplot of the intensities for the ChIP-seq peaks of H3K4me3. Intensities are plotted for the populations with different expression levels measured by RNA-seq and are indicated on the x-axis (Gene Expression Values). Asterisks indicate statistically significant differences, as evaluated by Wilcoxon’s singed rank test (P-values, ***P < 0.001). **(b)** Scatterplot representing the ChIP-seq peak signal intensities of H3K4me3 on the y-axis and gene expression values on the x-axis (n = 6,105). Pearson’s correlation co-efficiencies of the plots (R = 0.71) are also shown in the graph area. Dotted lines on the x-axis show 10 RPKM and y-axis show 1 × 10^4^ H3K4me3 intensities. Labels on each quadrant of the graph (e.g., ChIP (+) / RNA (−)) are the names given to these set of genes, and are used continually throughout this manuscript. **(c, d, e)** Graphical representation of the patterns of ChIP-seq (H3K4me3 and pol II) and RNA-seq data for the RSP8 gene **(c)**, PTGS2 gene **(d)** and NES gene **(e)**. Arrows indicate the direction of transcription and N/A indicates the lack of recognized peaks by MACS. Note that while the ChIP-seq of H3K4me3 and pol II consistently indicate active gene expression of RSP8 and PTGS2 genes, essentially no gene expression was observed for PTGS2 **(d)**. For the NES gene, RNA-seq tags were observed despite the lack of ChIP-seq signals of H3K4me3 and pol II.

We quantitatively analyzed the correlation between ChIP-seq and RNA-seq data for genes with ChIP-seq “peaks”. As shown in Figure 1a, we observed a positive correlation; mRNAs with higher expression levels were associated with higher ChIP-seq signal intensities, and we observed Pearson’s correlation of R = 0.71 (p-value < 2.2 × 10^−16^) with a log-transformed scatterplot (Figure 1b). When we examined individual genes, it was often possible to observe active transcription with large H3K4me3 ChIP-seq peaks, which is shown in Figure 1c. In contrast, we observed a number of cases in which RNA expression was insignificant, despite significant chromatin marks, as shown in Figure 1d. A significant population deviated from the straightforward expected distributions in Figure 1b. When we set the threshold of more than 1 × 10^4^ for H3K4me3 ChIP-seq intensity, presence of polII ChIP-seq and less than 10 RPKM for RNA expression, we identified 2,861, genes (ChIP (+)/RNA (−): upper left corner in Figure 1b) in which significant levels of ChIP-seq peaks and low levels of RNA-seq were detected, as shown in Figure 1d. In addition, for 2,897 genes (ChIP (−)/RNA (+): bottom right corner in Figure 1b), although ChIP-seq intensities were less than 1 × 10^−4^ for H3K4me3 and no polII ChIP-seq peaks were detected, RNA-seq indicated significant RNA levels of more than 10 RPKM, as shown in Figure 1e. Thus, we identified discrepancies between the ChIP-seq data and the RNA-seq data for a significant population of genes.

### Correlation among half-lives of the transcripts, chromatin marks and transcript levels

To examine the cause of the discrepancy, we focused on mRNA stabilities. We used our unique method, BRIC-seq, in which the nascent RNAs are labeled with 5′-bromouridine (BrU) and subjected to massive sequencing analysis in a time-lapse manner. By calculating the number of BrU-labeled RNA tags that remain in the population after a particular time duration, BRIC-seq can be used to measure each RNA half-life at a genome-wide level [21,22]. Detailed sequencing statistics for representative cases are shown in Additional file 1: Figure S1.

**Figure 2:**
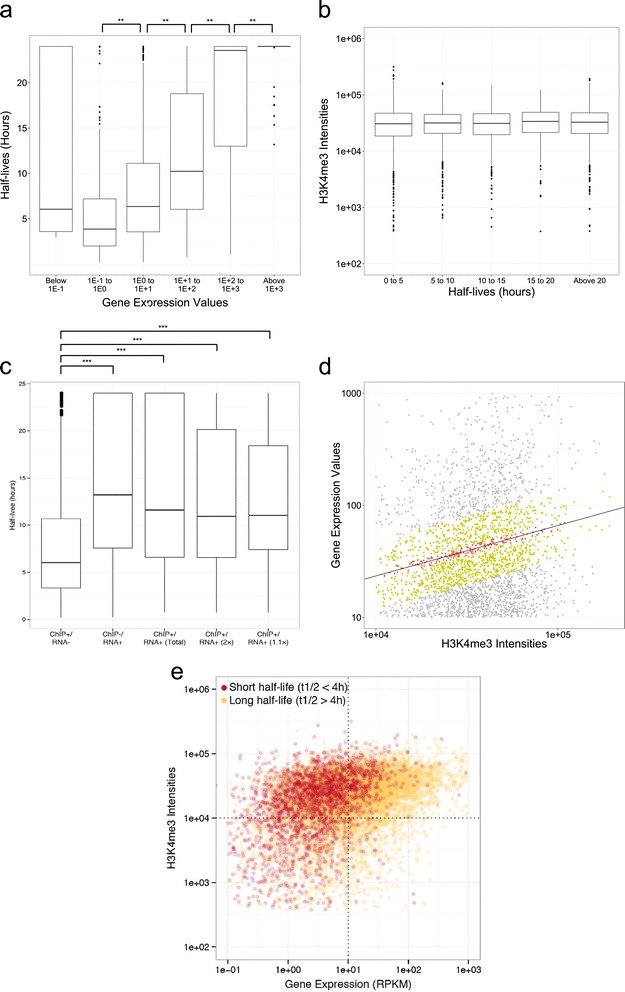
**Relationships between RNA half-lives, transcript levels and chromatin marks. (a)** Boxplot chart of the RNA half-lives of mRNAs of varying expression levels. **(b)** Boxplot charts of the signal intensities for the ChIP-seq peaks plotted for gene populations with different RNA half-lives, as indicated on the x-axis. **(c)** Boxplot chart of the half-life distributions in different ChIP-seq and RNA-seq fractions. Labels on the x-axis refer to the quadrant from Figure 1b. For ChIP (+) / RNA (+), ‘total’ indicates the total transcripts, and ‘×2’ and ‘×1.1’ indicates all the genes within 2-fold (green dots in Figure 2d) and 1.1-fold (red dots in Figure 2d) of the least squared regression line, respectively. Asterisks in **(a–c)** indicate statistical significance by Wilcoxon’s signed rank test (P-values *P < 0.05, **P < 0.01, ***P < 0.001). **(d)** Scatterplot to show the distributions of ChIP (+) / RNA (+) genes. Line is the least squared regression line. Red and green dots indicate genes within 1.1-fold and 2-fold of the least squared regression line, respectively. **(e)** Scatterplot representing the ChIP-seq peak signal intensities of H3K4me3 on the y-axis and gene expression values on the x-axis for transcripts with ChIP-seq peaks and measured half-lives (total n = 12,479). Genes having “short” (t_1/2_ < 4 h) half-lives (short n = 3190) are indicated in red dots and were statistically significant (p-value 6.8 × 10^−16^). Dotted lines show the 10 RPKM and 1 × 10^4^ peak intensities.

We examined the relationship between the eventual mRNA levels and the half-lives for genes with ChIP-seq “peaks”, which reflect active transcriptional initiation. In these cases, we observed positive correlation (Figure 2a), in which the half-lives of the transcripts were shorter in proportion to the decreasing expression levels. However, we detected no correlations between the half-lives of the mRNAs with the ChIP-seq intensities (Figure 2b). These results indicate that RNA stability may be a contributing factor for the determination of eventual transcript levels. Furthermore, the mRNA stability control is independent of transcriptional initiation, which is inferred by chromatin states.

Based on these observations, we speculated that control of the stability of mRNAs might play a pivotal role in determining the eventual RNA levels, particularly in case in which the ChIP-seq and RNA-seq data were inconsistent (ChIP (+)/RNA (−) population in Figure 1b); where, the transcript levels may be suppressed at a low level, despite their active transcription, owing to fast RNA turnover rates. To examine this possibility, we compared the half-lives of mRNAs between gene groups having H3K4me3 ChIP-seq intensities of more than 1 × 10^4^, a presence of polII ChIP-seq peak, with gene expression levels below 10 RPKM (ChIP (+)/RNA (−)) and those with H3K4me3 ChIP-seq intensities higher than 1 × 10^4^, with a presence of polII ChIP-seq peak and gene expression values above 10 RPKM (ChIP (+)/RNA (+)) or those with H3K4me3 ChIP-seq intensities lower than 1 × 10^4^, with a lack of polII ChIP-seq peak and gene expression values above 10 RPKM (ChIP (−)/RNA (+)), genes that half-lives could be measured. As shown in Figure 2c, we found that half-lives of the transcripts for ChIP (+)/RNA (−) genes were significantly shorter than those of ChIP (+)/RNA (+) genes (p value < 2.2 × 10^−16^) and ChIP (−)/RNA (+) genes (p value < 2.2 × 10^−16^). From the correlations between RNA-seq and ChIP-seq as shown in Figure 1b, it was possible to infer the gene expression levels from the ChIP-seq intensities for those genes in which the RNA-seq and ChIP-seq were consistent. When we examined genes for which gene expression values were within a 2 or 1.1 fold difference from those expected from the ChIP-seq intensities (Figure 2d), we observed narrower distribution of the mRNA half-lives with a median value of 11.0 and 10.9 hours, respectively (Figure 1c and Table 2). These half-lives of ChIP (+)/RNA (+) genes may serve as the default half-lives of those genes, and if genes do exhibit this particular mRNA half-life, the transcriptional initiation levels should be the major determinants of the eventual transcript levels.

**Table 2:** **Statistics of the half-life associated with ChIP (−) and ChIP (+) genes**

	**Total**	**ChIP(−) RNA(−)**	**ChIP(−) RNA(+)**	**ChIP(+) RNA(−)**	**ChIP(+) RNA(+) (Total)**	**ChIP(+) RNA(+) (**×**2)**	**ChIP(+) RNA(+) (**×**1.1)**
**Number of genes with a RNA half-life**	**12,479**	**6,235**	**603**	**2,745**	**2,896**	**1,617**	**187**
**Median half-life**	**nd**	**nd**	**13.2**	**6.0**	**11.6**	**11.0**	**10.9**

In Table 2, we only considered genes with compatible half-life measurements from BRIC-seq. ChIP(+): H3K4me3 intensities larger than 1 × 10^4^ and a presence of polII peak , ChIP(−): H3K4me3 intensities smaller than 1 × 10^4^ and an absence of polII peak. RNA(+): RPKM value larger than 10, RNA(−): RPKM value smaller than 10. For ChIP(+)/RNA(+) region; total: all genes, ×2: genes within two-fold of the regression line, ×1.1: genes within the 1.1-fold of the regression line. nd: not determined.

For further analysis into the correlation, we also selected mRNA with ‘short’ half-lives, specifically a total of 3,190 genes that had half-lives shorter than 4 hours. From the default half-life of 10.9 hours, we observed the standard deviation of the half-lives to be 3 hours, indicating that 4 hours was approximately the 95^th^ percentile confidence level. These particular mRNAs are highlighted as red dots in Figure 2e. We observed the enrichment of mRNAs with short half-lives in the ChIP+/RNA- fraction of the scatterplot (P-value = 6.8 × 10^−16^), in which gene expression values were lower than estimated by ChIP-seq data. These results suggest that control of mRNA stability is an important factor in determining the eventual mRNA levels for this population and indicate that the expression levels of these 866 genes may be controlled by mRNA stability in HeLa cells (see Additional file 2: Table S2). For validation, we used actinomycin D (ActD), a transcriptional inhibitor, and chased the RNA decay by RT-qPCR (Additional file 1: Figure S2). BAMBI and MED26 RNA half-lives (defined in BRIC analysis as ChIP(+)/RNA(−)/short RNA half-life) determined by BRIC analysis were similar to those determined by ActD analysis. The transcripts of MMP2 and SLC25A23 (defined in BRIC analysis as ChIP(−)/RNA(+)/long RNA half-life) were determined as stable RNAs in both BRIC and ActD chase analyses. In contrast, the RNA half-lives of ZNF691 and ZNF574 slightly varied between BRIC and ActD chase analyses. Thus, most RNA stabilities determined by BRIC analysis were confirmed by ActD chase analysis.

To analyze the categories of genes that receive regulation at either transcriptional initiation or RNA half-life levels, we ran GO enrichment analysis. Among the ChIP (+)/RNA (+) genes, GO terms associated with basic translation or transcriptional machineries were enriched (Table 3a). For the ChIP (−)/RNA (+) genes, genes associated cytoplasm as a location were enriched (Table 3b), and for the ChIP (+)/RNA (−) genes, GO terms associated with transcription factors were enriched, particularly among genes with a short half-life (t_1/2_ < 4 h) (Table 3c-d). These results suggest that different functional categories of genes are subjected to different modes of gene expression regulation.

**Table 3:** **List of GO enrichments for transcripts in different ChIP-seq and RNA-seq fractions**

**A) ChIP (+)/RNA (+) GO enrichment**			
**GO:ID**	**GO: term**	**Number of genes**	**False-discovery rate**
**GO: 0044822**	**poly (A) RNA binding**	**618**	**6.00e-261**
**GO: 0006412**	**translation**	**293**	**3.86e-196**
**GO: 0010467**	**gene expression**	**387**	**5.61e-162**
**B) ChIP (−)/RNA (+) GO enrichment**			
**GO: ID**	**GO: term**	**Number of genes**	**False-discovery rate**
**GO: 0005737**	**cytoplasm**	**138**	**2.26e-2**
**GO: 0070062**	**extracellular vesicular exosome**	**74**	**2.61e-2**
**GO: 0005635**	**nuclear envelope**	**15**	**2.81e-2**
**C) ChIP (+)/RNA (−) GO enrichment**			
**GO: ID**	**GO: term**	**Number of genes**	**False-discovery rate**
**GO: 0003677**	**DNA binding**	**378**	**2.96e-28**
**GO: 0006355**	**regulation of transcription, DNA-templated**	**266**	**4.44e-17**
**GO: 0006351**	**transcription, DNA-templated**	**361**	**8.60e-17**
**D) ChIP (+)/RNA (−)/half-life < 4 h GO enrichment**			
**GO: ID**	**GO: term**	**Number of genes**	**False-discovery rate**
**GO: 0003677**	**DNA binding**	**216**	**5.19e-58**
**GO: 0006351**	**transcription, DNA-templated**	**192**	**1.37e-37**
**GO: 0006355**	**regulation of transcription, DNA-templated**	**147**	**5.13e-34**

### Identification of UPF1, EXOSC5 and STAU1 as controlling factors for RNA stabilities

**Figure 3:**
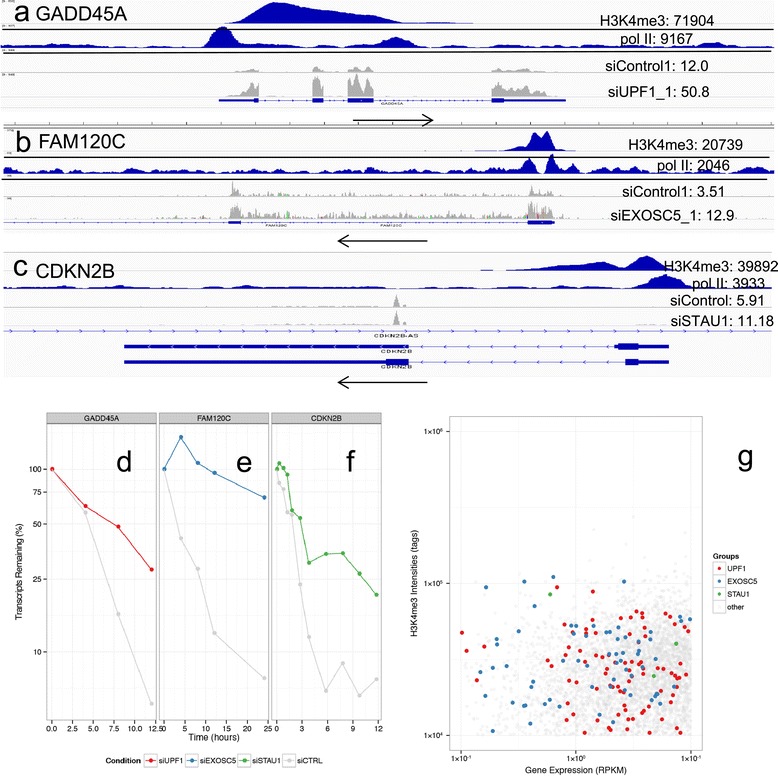
**Identification of UPF1, EXOSC5 and STAU1 as factors controlling RNA half-lives. (a–c)** ChIP-seq and RNA-seq for the GADD45A **(a)**, FAM120C **(d)** and CDKN2B **(c)** genes, which showed significant changes in RNA half-lives in UPF1, EXOSC5 and STAU1 knocked-down cells, respectively. Upper two panels in **(a–c)**: ChIP-seq data for H3K4me3 and pol II. Lower two panels in **(a–c)**: RNA-seq data for control cells and the respective knockdown cells. **(d–f)** BRIC-seq normalized graph for GADD45A **(d)**, FAM120C **(e)** and CDKN2B **(f)** genes in control cells or cells knocked-down for UPF1 **(d)**, EXOSC5 **(e)**, and STAU1 **(f). (g)** Scatterplot representing the ChIP-seq peak signal intensities of H3K4me3 on the y-axis and gene expression values on the x-axis with putative UPF1-controlled genes, EXOSC5-controlled and STAU1-controlled genes in blue, green and red dots respectively.

To evaluate the potential contribution of known RNA degradation factors to the control of global RNA stability, we chose three representative factors for analysis: UPF1, EXOSC5, and STAU1. It has been reported that UPF1 regulates 3–20% of transcripts [11-13], highlighting the potential importance of UPF1 in regulating RNA degradation and abundance. EXOSC5 is an essential component of the exosome complex that is the major mRNA degradation machinery in mammalian cells. To analyze the alteration of global mRNA turnover by perturbation of representative factors, we examined EXOSC5. STAU1, which regulates around 1% of *bona fide* mRNAs [18], is a typical RNA-binding protein involved in RNA degradation. We used data from BRIC assay in the cells depleted in UPF1 by siRNA (see Methods for accession numbers). As observed in a previous study [23], mRNA levels of the GADD45A gene, which is a known target of UPF1, were increased following UPF1 knockdown (Figure 3a), with an increase in half-life (Figure 3d). We then conducted similar experiments using EXOSC5 and STAU1 knockdown cells and prepared a similar RNA-seq and BRIC-seq dataset. We observed an increase of FAM120C mRNA levels (Figure 3b) and an increase in half-life (Figure 3e) in EXOSC5 knockdown cells. In STAU1 knockdown cells, the mRNA levels of CDKN2B were increased (Figure 3c), with increased half-lives (Figure 3f).

We next looked for genes that may be regulated by these factors, and identified 266, 219 and 39 genes where the mRNA half-lives were extended by more than two-fold (in UPF1 and EXOSC5 knockdown cells) or 1.5-fold (in STAU1 knockdown cells) and showed mRNA expression increase by two-fold (in UPF1 and EXOSC5 knockdown cells) or 1.5-fold (in STAU1 knockdown cells) in UPF1, EXOSC5 and STAU1 knockdown cells, respectively. As shown in Figures 3a, 3b and 3c, we noticed that the transcripts that were not observed in the control knockdown cells appeared in the knocked-down cells in many cases. We examined the distribution of the dots of these genes, whose transcripts were stabilized and increase in corresponding knockdown cells, in Figure 1b and found that they are enriched in the upper-left corner of the plot. In total, we identified 23, 40 and 4 genes (Additional file 2: Table S3) whose mRNA half-lives are potentially controlled by UPF1, EXOSC5 and STAU1 respectively, consisting of 3, 5, and 0.5% of the total of 1,279 genes (ChIP (+), RNA (−), half-life < 4 h) in this area. We also examined the overlap between the genes controlled by UPF1 and EXOSC5 and found little overlap (Table 4). Although we could identify UPF1, EXOSC5 and STAU1 as control factors only for a limited population (8% of ChIP+/RNA-/t1/2 < 4 h genes) by this approach. These observations are only the first step in identifying the role of RNA decay factors in determination of RNA abundance through RNA degradation, and further systematic analyses may facilitate identification of the complex regulatory mechanisms of mRNA stabilities.

**Table 4:** **Summary statistics used for the analysis of RefSeq transcripts**

**Conditions (Refseq)**	**Number of genes**
**Active transcription (supported by H3K4me3 and pol II peaks) and half-life measured**	**6,105**
**With short half-life (~4 h)**	**1,291**
**with low expression (10rpkm and below) and high H3K4me3 (1** × **10** ^**4**^ **and above)**	**866**
**and UPF1 target**	**26 (3.0%)**
**or EXOSC5 target**	**40 (4.6%)**
**or STAU1 target**	**4 (0.46%)**

### Computational modeling of the effect of mRNA half-lives on eventual mRNA levels

**Figure 4:**
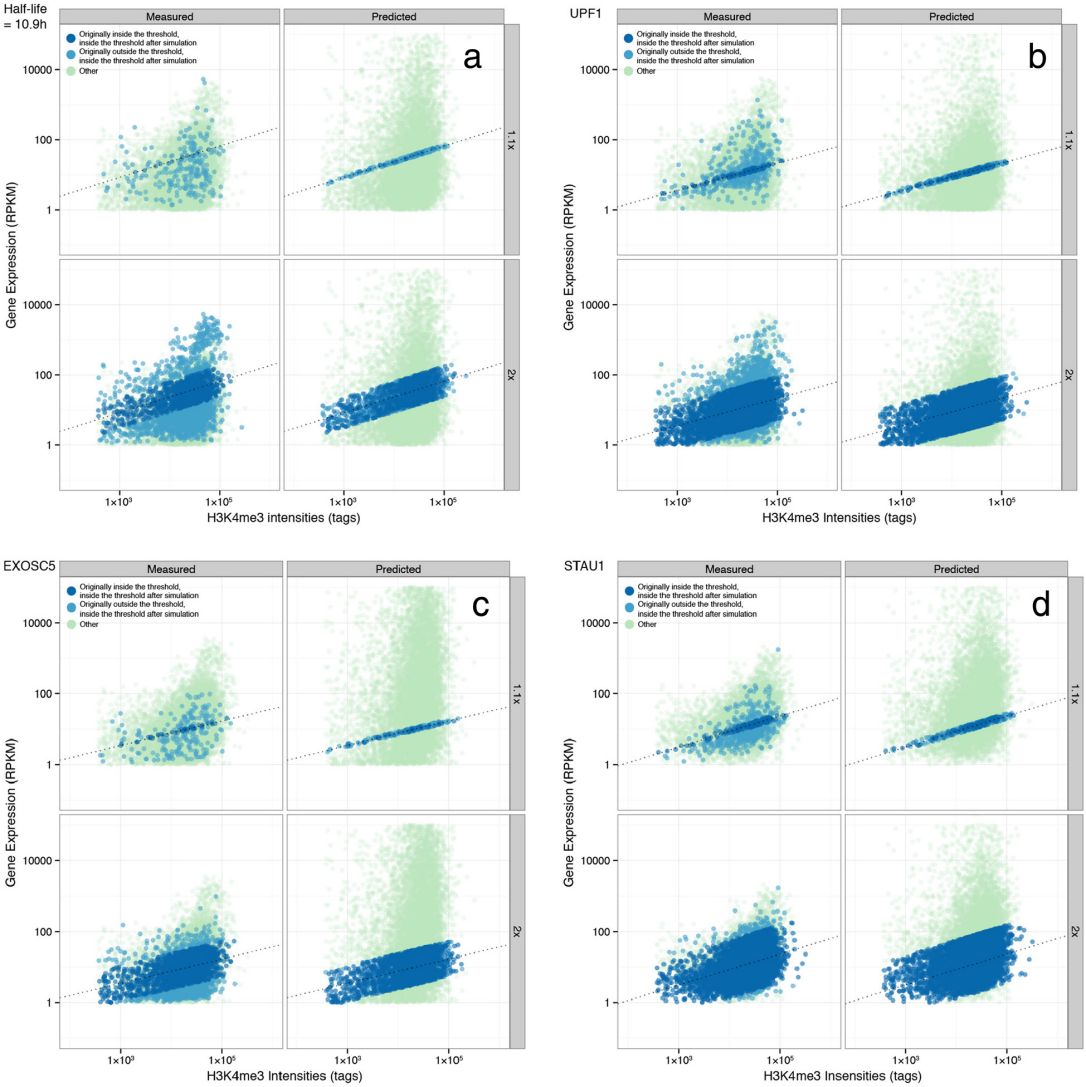
**Computational simulations of the RNA half-lives as a determinant for transcript level. (a)** Computational simulation into the effect of RNA half-life on RNA abundance. Each gene was simulated to have a half-life of 10.9 hours, potential default half-life, and the predicted gene expression was plotted against the H3K4me3 intensities, along with the measured gene expression. The dotted line indicates the least-squares regression line between the predicted gene expression and H3K4me3 intensities. **(b-d)** Result of the computational simulation of the predicted RNA expression when each RNA abundance was predicted from RNA half-life when **(b)**UPF1, **(c)** EXOSC5 and **(d)** STAU1 were knocked-down, in comparison to each control knock-down conditions. The predicted gene-expression of the decay factor knock-down and the measured gene-expression of control knock-down were plotted against H3K4me3 intensities. The dotted line indicates the least-squares regression line between the predicted gene expression and H3K4me3 intensities.

We predicted the RNA abundance by normalizing the half-life to be 10.9 hours, the estimated default half-life (from Figure 2c), and obtained the least squares regression line between the predicted RNA levels and H3K4me3 intensities. We made a threshold of × 1.1 and × 2, above and below the least-square regression line to define genes where the H3K4me3 intensities and RNA abundance correlate. We found that out of 9,407 genes that were available from BRIC-seq dataset, we found 2,593 and 242 genes that resided within × 2 and × 1.1 of the regression line, respectively (Figure 4a and Table 5). We checked the original gene expression of those genes prior to the simulation, and checked whether their measured RNA abundance correlates with H3K4me3 intensities. We found out of 2,593 and 242 genes that resided within × 2 and × 1.1 of the regression line from predicted gene expression, we found 1,540 and 229 genes where measured to be outside of the threshold, respectively. It means that the RNA stability of those genes contributed to the RNA abundance. Taken together, these results collectively support our claim that RNA degradation significantly contributes in determining the eventual expression levels (Figure 4a).

**Table 5:** **Summary statistics of computational simulation**

**Condition**	**t1/2=10.9 hours**	**siUPF1**	**siEXOSC5**	**siSTAU1**
Total	9407	9387	9852	9334
x2 predicted under condition (x)	2593	5408	2484	3753
of which was not within x2 in “measured”	1540	815	680	294
x1.1 under condition (x)	242	555	245	651
of which was not within x1.1 measured	229	439	200	486

Additionally, we conducted ChIP-seq on H3K27ac, H3K27me3, H3K36me3 to build a linear model as described by Wan*g*, C., *et. al.* [7]*,*. We built one linear model incorporating H3K4me3, H3K27Ac, H3K27me3 and H3K36me3 intensities with half-life as an extra variable, and one without the half-life, to explain the RNA abundance. We found that fitting increased from 0.41 to 0.58 in R-value, which confirmed the previous finding by Wang. *et. al.,*[7]*.*

We examined whether the changes in the RNA half-lives from knockdown of UPF1, EXOSC5 or STAU1 could explain the changes in the eventual transcript levels. For this analysis, we conducted a computational simulation. As shown in Additional file 1: Figure S3, we found that the described theoretical model can predict the changes of eventual RNA levels with Pearson’s correlation co-efficiency of 0.8, 0.8 and 0.7, respectively. Overall, we demonstrate that the simple computational model could reasonably explain the changes of eventual mRNA levels, thus supporting our idea that the major determinant of the eventual RNA levels in these cases is at the level of RNA stability. We further simulated the RNA abundance, from the changes in RNA half-life, in relation to the ChIP-seq signal levels and we found 439, 486, and 200 genes that were within × 1.1 of the regression line that lied outside of the threshold prior to simulation, for UPF1, EXOSC5, and STAU1, respectively (Figures 4b-d).

### Possible feedback between mRNA turnover and transcriptional initiation

To analyze the possible feedback mechanisms between mRNA turnover and transcription initiation, we used the genes in which both mRNA half-lives and eventual transcript levels were increased more than two fold in knockdown cells. In 975 and 6,309 genes in UPF1 and EXOSC5 knockdown cells, respectively, there were no significant changes in eventual transcript levels (within two fold) observed despite remarkable changes to their RNA half-lives (more than two fold). We speculated that there might be a possible feedback between mRNA turnover and transcriptional rate. If mRNAs for a particular group of transcriptional repression factors are included in the UPF1/EXOSC5 targets and their stabilized mRNAs result in increased protein levels of such transcriptional repression factors, thereby enhancing the repression activities on their target genes, it would explain unchanged balance of eventual transcript levels for these genes. We examined whether any transcription factor binding sites were enriched in the upstream regions of the 975 and 6,309 genes. In the case of UPF1, we detected significant enrichment of the consensus binding site for HIC1, which is a transcription factor belonging to the zinc finger family. We validated the changes of its expression level and half-lives in control and UPF1 knockdown cells, and confirmed HIC1 as a UPF1 target (Additional file 1: Figure S3a). We were unable to further validate direct binding of HIF1 to target genes, since no effective antibodies are available. Also, it is possible that HIC1 may not be the only candidate, which may contribute to the feedback regulation. Many zinc finger family transcription factors share consensus binding sequences. The list of putative zinc finger family transcription factors that have significant homology to HIC1 in their DNA binding domains with extended half-lives (e.g. ZNF783 shown in Additional file 1: Figure S3b) and increased eventual transcript levels upon UPF1 knockdown are shown in Additional file 2: Table S3. These factors may collectively enable elaborate regulation of gene expression.

### Identification of candidate genes controlled through RNA stability in other cell types

To further extend our idea that controls at the level of mRNA decay contribute to determining eventual mRNA expression levels in other cell types, we analyzed the published ENCODE data [25] and DBTSS [26], which included ChIP-seq data of H3K4me3 and pol II and RNA-seq in a wide variety of cell types. In addition, the RNA-seq data of subcellular fractionated mRNAs were included in the dataset. We selected eight cell types for which all these datasets were available (details in Additional file 1: Figure S4a). We retrieved and analyzed the ChIP-seq data and RNA-seq data as performed with HeLa cells. First, as shown in Additional file 1: Figure S6, we observed weak correlations between ChIP-seq intensities of H3K4me3 and gene expression values as reported in HeLa cells.

**Figure 5:**
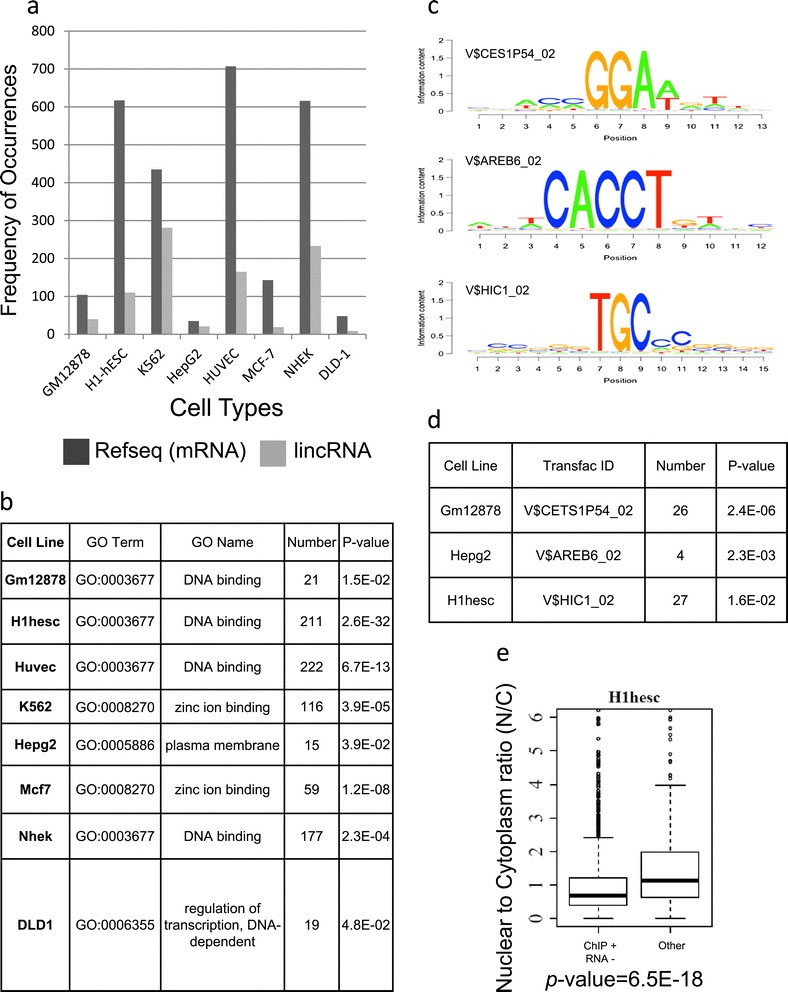
**Screening of candidate genes that may be controlled at the level of RNA degradation from the ENCODE dataset. (a)** Number of candidate genes screened from the indicated cell types based on the ENCODE data. **(b)** Gene ontology (GO) enrichment of genes in the ChIP (+) / RNA (−) regions in the indicated cell types based on the ENCODE data. ChIP-seq signal intensities were comparable between different cell types, although RNA-seq-based gene expression values were remarkably different. The GO term enrichment with the lowest P-value for each cell line is shown. **(c, d)** Enriched consensus transcription factor binding sites for genes in the ChIP (+) / RNA (−) region for ENCODE dataset. **(c)** Graphical representation of the enriched consensus binding sites in the promoter regions of the genes in ChIP (+) / RNA (−) region for ENCODE dataset. **(d)** The list of cell lines with consensus binding site enrichment from the TRANSFAC database. **(e)** The nuclear/cytoplasmic gene expression values for the genes where RNA half-life may be the contributor to the RNA levels and all other genes in H1 human embryonic stem cells (hesc). Statistical significance of the difference is indicated under the x-axis.

Since there were no BRIC-seq data in the ENCODE dataset, we could not directly analyze the genes with short mRNA half-lives. Nevertheless, we could select genes for which ChIP-seq tags of H3K4me3 and pol II were associated, thus indicated as actively transcribed in the cell line, although their gene expression levels were not at the expected levels. In the ENCODE dataset, we also considered a positive signal of H3K36me3, which is a chromatin mark for transcriptional elongation, to further assure active transcription [27]. We identified an average of 338 candidate genes with active transcription and low RNA abundance in each cell type (Figure 5a), which should be regulated at the level of mRNA decay. We examined and identified GO terms that were significantly enriched, depending on cell types, and the top enriched terms were predominantly associated with DNA binding (Figure 5b). We also analyzed whether there are any cases for a particular gene to be selected as such a candidate in a cell type-preferred manner. We found that 2,705 potential controls at the level of RNA half-life were observed in a single cell type (Additional file 2: Table S5, Additional file 1: Figure S6). We also examined if there was possible feedback between controls of RNA half-lives and transcription initiations. We found that several transcription factor binding consensus sequences of the ETS family and AREBP family genes are enriched in the promoters of the genes inferred to be regulated at the level of RNA stability in Gm12878 cells and HepG2 cells, respectively. In addition, we identified HIC1 binding consensus sequences in the promoters of the genes inferred to be regulated at the level of RNA stability in human H1 embryonic stem cells. The feedback regulations may be common in various cell types with distinct responsive transcription factors depending on cell type (Figures 5c, d). We further analyzed the subcellular localizations of the mRNAs of these genes using the corresponding ENCODE data. We found significantly enriched mRNAs in the cytoplasm only in the embryonic stem cells (H1-hES) (*p*-value 6.5 x 10^−18^) (Figure 5e), suggesting there may be a characteristic regulatory mechanism for controlling RNA stability in the cytoplasm of ES cells. On the whole, these data should provide an important complement to the ENCODE annotations, which aim to generate a complete catalogue of genetic elements explaining gene expression regulation.

### Distinct controls of the RNA stabilities of mRNAs and non-coding RNAs

**Figure 6:**
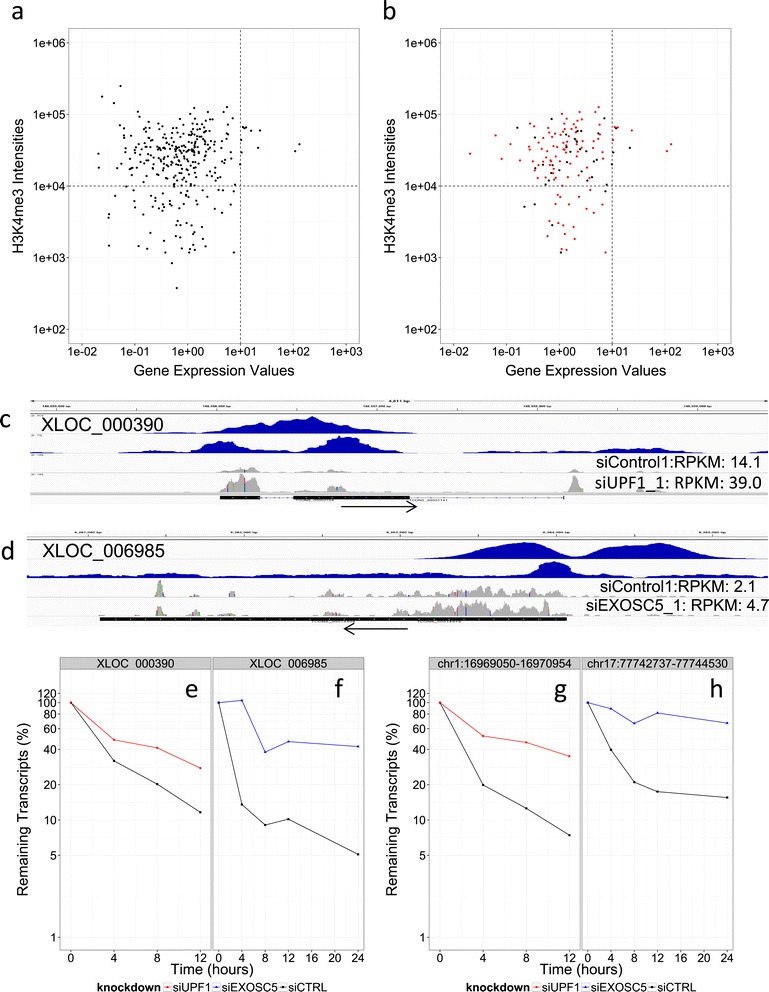
**Contribution of the RNA half-lives to transcriptional regulations of lincRNAs. (a, b)** Scatterplot showing the relationships between H3K4me3 intensities and gene expression for all lincRNA **(a)** and those that were BRIC-seq compatible (red dots: t_1/2_ < 4 h) **(b). (c, d)** Examples of lincRNAs regulated by UPF1 **(c)** and EXOSC5 **(d)**. Upper panels are H3K4me3 and pol II peaks, and lower panels are RNA-seq measurements for the control and indicated knockdown. UPF1 **(e)** and EXOSC5 **(f)** show the normalized decay curve from the BRIC-seq measurements for these examples. Red lines are siUPF1 **(e, g)** or siEXOSC5 **(f, h)** data, respectively and blue lines are si_control data. Enhancer RNAs (eRNAs) regulated by UPF1 **(g)** or EXOSC5 **(h)**. The label shows the genomic coordinates of these eRNAs (in hg19 build). These show normalized decay curves for the BRIC-seq measurements for these examples as above.

To examine whether the regulations at the level of RNA half-lives are observed in lincRNAs, we conducted a similar analysis for lincRNAs in HeLa cells, as shown in Figures 1 and 2. We tentatively defined the dataset of lincRNA as that of lincDB [28]. As shown in Figures 6a and 6b, we associated the ChIP-seq peak intensities and gene expression values. We unexpectedly observed distinct patterns from those of mRNAs. Namely, among 141 lincRNAs in HeLa cells, 103 lincRNAs had “short (<4 h)” RNA half-lives. Of these, 84 (82%) resided in the upper-left corner of the plot, suggesting that most of the lincRNAs are controlled at the post-transcriptional level. Because there are reports on the possible involvement of NMD in regulating non-coding RNA [29,30], we examined the possible involvement of UPF1 in the regulation of lincRNAs. Interestingly, none of the 84 lincRNAs were detected as potential UPF1-controlled transcripts. In this data set, UPF1 may have only limited contribution. By contrast, we found 26 (31%) of the lincRNAs (Table 6, Additional file 2: Table S3) were regulated by EXOSC5.

**Table 6:** **Summary statistics used for the analysis of lincRNAs**

**Conditions (lincDB)**	**Number of genes**
**Active transcription and half-life measured**	**141**
**With short half-life (~4 h)**	**103**
**with low expression (10RPKM and below) and high H3K4me3 (1E4 and above)**	**84**
**and UPF1 target**	**0**
**or EXOSC5 target**	**26 (31%)**

We next examined whether enhancer RNAs (eRNA), which facilitate the functions of the enhancers, are also regulated at the level of RNA degradation. We again used the ENCODE data from HeLa cells. We retrieved the ChIP-seq data of H3K4me and H3K27Ac, which are representative chromatin marks of active enhancers. Among 49,903 genomic regions having “peaks” of both H3K4me1 and H3K27Ac, we identified 77 cases in which there were RNAs in the overlapping regions (shown in Figure 6g), 1.5 kb away from any RefSeq gene body and their RNA half-lives were extended by UPF1 knockdown by more than two fold in BRIC-seq assay. Similarly, we identified 358 cases in which half-lives of RNAs in overlapping regions were examined (shown in Figure 6h, listed in Additional file 2: Table S6) and in which transcripts showed extended RNA half-lives of more than two-fold in EXOSC5 knockdown cells. Although further detailed experimental validations are necessary, these results may indicate that controls mediated by RNA stability are used in determining the transcript levels of non-coding RNAs.

## Discussion

Here, we have described a genome-wide correlation among the signal intensities of ChIP-seq, gene expression values measured by RNA half-lives measured by BRIC-seq. We identified that regulation at the level of RNA degradation plays an important role in determining eventual RNA levels. We demonstrated that this control may exhibit a particularly large effect in cases in which the ChIP-seq data and RNA-seq data are inconsistent. Indeed, in this study, we estimated that the abundance of 866 mRNAs is regulated by RNA degradation in HeLa cells. Furthermore, we applied similar approaches to analyze the public ENCODE data and identified a total of 2,705 candidate genes whose gene expression levels are likely to be controlled at the level of RNA stability. We also found that these controls appeared to vary among cell types. To our knowledge, this is the first report that describes the integration of the ChIP-seq, RNA-seq and RNA half-life data in identifying genes that may receive post-transcriptional gene expression regulation. In GO analyses, there are some limitations because GO terms may be loosely defined for a particular gene. However, we first selected GO terms with statistical significance (FDR < 0.05), and we only used GO term enrichment with more than 10 genes in a set to ensure minimum false positives, which will ensure that the GO terms described are representative of the biological phenomenon. We then found enrichment of transcription factors in transcripts with discrepancies between the ChIP-seq and RNA-seq with short half-lives, and in particular, negative regulatory factors. In a recent study, Haimovich *et al.* [31] indicated that some RNA degradation factors play a role in transcription, implicating a feedback loop for gene expression. Our data suggest another mechanism by which this occurs, through a faster degradation rate of mRNAs encoding transcription factors, particularly for those that negatively regulate gene expression, thus affecting the eventual RNA levels. Previous studies conducted by Wang *et. al.*, demonstrated that RNA stability could in inferred from the residual errors in modeling the RNA abundance from ChIP-seq data and they validated their claims from a half-life data; however, they used RNA stability data from a different cell-line [7], and they could not accurately predict the eventual RNA abundance from the RNA stability. Herein, by analyzing RNA stability data from the same cell-line as RNA-seq data, we were able to estimate the contribution of the RNA stability to the RNA abundance. In addition we were able to estimate the RNA stability contributions on the RNA abundance upon UPF1, STAU1 and EXOSC5 knockdown.

Although we did not demonstrate how the RNA half-lives are controlled in the current study and we identified UPF1, EXOSC5 and STAU1 as control factors in some cases, they could explain at most 8% of the total mRNA population. Even for the cases of candidate UPF1, EXOSC5 or STAU1-controlled genes, it is possible that they may not be direct targets of these factors and that we may have picked up secondary or later effects as a consequence of UPF1, EXOSC5 or STAU1 knockdowns. Another obvious drawback of our approach is that mRNA half-lives were not directly measured by BRIC-seq for the ENCODE dataset. Therefore, it is possible that they may be mediated by other regulatory mechanisms, rather than at the level of RNA half-lives, such as RNA halting and abortive transcriptions. To minimize these possibilities, we selected the cases in which H3K36me3, a marker of transcriptional elongation, should be significant in the transcript regions.

In spite of several drawbacks, we believe that genome-wide features of correlation among ChIP-seq, RNA-seq and BRIC-seq should give an important starting point to further explore posttranscriptional regulatory mechanisms, for which only limited knowledge has been accumulated. Indeed, recent papers have begun to reveal many human diseases that are caused by malfunctions of RNA decay pathway. In particular, it has been made gradually clear that most immune-response mRNAs are destabilized when they are not required via their cis-regulatory elements in the 3′ UTR. It is proposed that such RNA-decay mechanisms collectively enable rapid up-/downregulation of gene expression in response to environmental changes. The AU-rich element (ARE) is one of such elements widely found in the 3′ UTR of mRNA of immune-related genes. Mice lacking ARE in the TNF-alpha mRNA showed joint and gut-associated immunopathologies [32]. The trans-acting factor regulatory RNase 1 (Regnase-1, also known as Zc3h12a or MCPIP1), which is induced by Toll-like receptor (TLR) ligands, interleukin (IL)-1β and MCP-1, is involved in the destabilization of mRNAs including Il6 mRNA. Regnase-1-deficient mice develop severe autoimmune disease because of excess production of cytokines [33], highlighting the importance of RNA degradation-mediated gene regulation. In addition to immunological disorders, there are a growing number of cases with impaired RNA decay regulation that cause disease, and they sometimes reveal unexpected connections between otherwise completely unrelated diseases. Perlman syndrome, an autosomal recessively inherited congenital overgrowth syndrome associated with high neonatal mortality, is an obvious example. The survivors of this disease have a high risk of Wilms tumor. Recently, it was reported that the responsible gene of this disease is the exoribonuclease DIS3L2, a homologue of exosome component DIS3 [34,35]. Moreover, DIS3L2 is mutated in approximately 3–6% of carcinomas [35].

In this study, we have also analyzed the stabilities of non-coding RNAs (lincRNAs and eRNAs) and mRNAs. We and another study reported that stability of non-coding RNAs is also tightly regulated, suggesting that the instability contributes to the dynamic nature of lincRNAs [22,36]. Indeed, the stability of noncoding RNAs has an impact on their biological function [29,37-39], although the exploration into its relevance in human disease has just begun. Further enrichment of our knowledge on the control mechanisms on RNA stability both for mRNAs and non-coding RNAs will shed new light on putative disease-associated genetic or somatic mutations.

## Conclusions

By integrative analysis of ChIP-seq, RNA-seq and our BRIC-seq, we showed that RNA half-life may serve as an important post-transcriptional determinant of gene expression. We suggest that UPF1, EXOSC5 and STAU1 may play active roles in such controls. In addition, we propose the linkage between transcription and RNA decay through regulated degradation of mRNAs encoding transcription factors to maintain the steady state level of RNA abundance.

## Methods

RNA-seq and BRIC-seq data for UPF1 were obtained from a previous study [23]. The accession numbers for the sequencing data are [DDBJ:DRA000591] and [DDBJ:DRA001215]. ‘Basal’ RNA-seq libraries, EXOSC5 and STAU1 knockdown RNA-seq libraries were sequenced according to the standard protocol from mRNA-seq Sample Preparation (Illumina, San Diego, CA). The outline of the experimental procedures is as follows.

### Cell culture and siRNA transfection

HeLa cells were grown in Dulbecco’s Modified Eagle’s Medium (DMEM) supplemented with 10% fetal bovine serum and antibiotics at 37°C at 5% CO_2_ in a humidified incubator. siRNAs were transfected (final concentration 10 nM) using Lipofectamine RNAiMAX (Invitrogen, Carlsbad, CA), according to the instructions from the manufacturer. Cells were harvested 72 h after the transfection. The knockdown efficiencies were determined by RT-qPCR (see Additional file 1: Figure S7). The sequences of siRNAs are provided in Additional file 1: Figure S8.

### RT-qPCR

The isolated RNAs were reverse-transcribed into cDNA using the PrimeScript RT Master Mix (TaKaRa, Otsu, Japan). The target cDNAs were amplified by SYBR Premix Ex Taq II (TaKaRa) according to the manufacturer’s instructions, using the primer sets listed in Additional file 1: Figure S8. GAPDH was used for normalization. Quantitative real-time reverse transcription PCR analysis was performed using a Thermal Cycler Dice Real Time System (TaKaRa).

### RNA-seq

Approximately 1 μg RNA was used to sequence an RNA-seq library using the mRNA-seq Sample Preparation Kit (Illumina) according to the manufacturer’s protocol. Thirty-six base pair single-end-read RNA-seq were generated from the Illumina GA sequencer, according to the standard protocol. The fluorescent images were processed to nucleotide sequences using the analysis Pipeline software supplied by Illumina. The reads mapping to the ribosomal RNA genes were removed. The filtered sequences were mapped to the reference human genome (hg19) using Tophat (version 2.0.8) [40], only allowing the reads to be processed if the reads were compatible with the gene annotation files from the RefSeq [41] and lincRNA [28] databases (downloaded on 2^nd^ July 2013). For the enhancer RNA (eRNA) analysis, Tophat (version 2.0.8) was used but without specifying the annotation and allowing novel splice-junctions to occur. Mapped reads were quantified using Cufflinks (version 2.1.1) [42]. The transcript with the highest expression was used as a representative transcript for the given gene and the RPKM values of all transcripts in the same genes were added together to give RPKM values for the gene.

### BRIC-seq

BRIC was performed as previously described [21,22]. In brief, cells were incubated at 37°C in the presence of 150 μM 5′-bromo-uridine (BrU) (Wako, Osaka, Japan) for 24 h in a humidified incubator with 5% CO_2_. After replacing BrU-containing medium with BrU-free medium, cells were harvested at indicated time points. Total RNA was isolated using RNAiso Plus (TaKaRa). Twelve micrograms of BrU-labeled total RNA were denatured by heating at 80°C for 1 min and then added to anti-BrdU mAb-conjugated beads containing 2 μg of anti-BrdU mAb (clone 2B1, MBL). The mixture was incubated at room temperature for 1 h with rotation. Beads were washed four times with 0.1% BSA in PBS. ISOGEN LS (Nippon Gene, Tokyo, Japan) was added, followed by RNA isolation, according to the manufacturer’s instructions. The isolated RNA was used for deep sequencing using the mRNA-seq Sample Preparation Kit using the same protocol as RNA-seq. Data processing was conducted by the identical procedures as the RNA-seq method above. For BRIC-seq data without transfection, we used 13 time points to calculate half-life: 0 min, 15 min, 30 min, 45 min, 1 h, 1.5 h, 2 h, 3 h, 4 h, 6 h, 8 h, 10 h and 12 h. For UPF1 knockdown data, four time-points were taken: 0 min, 4 h, 8 h and 12 h. For STAU1 knockdown data, 11 time-points were taken: 0 min, 15 min, 45 min, 75 min, 105 min, 165 min, 225 min, 345 min, 465 min, 585 min and 705 min. For EXOSC5 knockdown data, five time-points were taken: 0 min, 4 h, 8 h, 12 h and 24 h. Calculation of RNA half-lives were conducted as previously described [21].

### ActD chase analysis

Total RNA was isolated from HeLa cells at the indicated time points after addition of ActD (2 μg/ml), followed by RT-qPCR analysis to determine the degradation kinetics of each mRNA.

### ChIP-seq

ChIP-seq was conducted as previously described [43-45]. The following antibodies were used in each experiment: anti-histone H3K4me3 antibody (Abcam, Cambridge, UK, ab1012) ,anti-RNA polymerase II (Abcam ab817), monoclonal anti-H3K27me3 antibody (Abcam, ab6002), polyclonal anti-H3K27Ac antibody (Abcam, ab4729), polyclonal anti-H3K36me3 antibody (Abcam, ab9050) For ChIP-seq, Illumina’s Eland was used to map the 36 bp reads to the reference human genome (hg19). Peaks were called by MACS 1.4.1 [24] at default settings.

### Bioinformatic analysis

To assign a ChIP-seq peak to each gene, representative transcripts, defined by Cufflinks on the RNA-seq data without any transfection, were used and defined as a peak where there is an overlap by more than 1 bp between 1.5 kbp upstream and 1.5kbp downstream of the transcription start site (TSS). The number of tags per peak was calculated using the wig files generated from MACS and adding all tags in the peak region. For the RefSeq mRNA, we analyzed a total of 6,104 genes for which a peak was observed for both H3K4me3 and RNA polymerase II and for which a positive half-life could be calculated. Wilcoxon’s signed ranked test was used to determine the statistical significance between the bins of gene expression and H3K4me3 tags, the bins of RNA half-life and H3K4me3 tags, and the bins of gene expression and RNA half-life. Pearson product–moment coefficient was used to calculate the correlation values between log-transformed H3K4me3 tags and log-transformed gene expression. Gene ontology was conducted by R, obtaining the gene ontology database from NCBI, calculating the occurrence of a particular gene ontology (GO) term, followed by calculating the enrichment of a particular GO term in the sample gene-list by hyper-geometric distribution, corrected for multiple testing by Benjamini-Hochberg false-discovery rate. GO data was obtained on 8^th^ May 2014. To define eRNAs, we used H3K4me1 and H3K27Ac HeLaS3 data from the ENCODE project. Bedtools [46] were used to identify and quantify the mapped reads from siUPF1/siEXOSC5 and siControl BRIC-seq dataset that maps to the H3K4me1 and H3K27Ac regions and not overlapping with 1.5 kb of the entire length of the gene body. The number of mapped reads to a particular region was normalized by the length of the region (to 1 kbp) and by the sequencing depth (to per million). The reads were normalized by GAPDH and eRNA half-lives were calculated as above.

### Computational simulation and modeling

The ChIP-seq data was analysed as previously mentioned for H3K4me3 and pol II, and the intensities were calculated by counting the number of tags mapped within: +/− 1kbp window centered on the TSS for H3K4me3 and, H3K27Ac, and gene body for H3K27me3 and H3K36me3. The log-transformed and standardized (mean = 0 and standard deviation = 1) histone intensities were used to build a linear model [7].

$$ \mathrm{Model}\ \mathrm{A}:\ \mathrm{mRNA}\ \mathrm{level} \sim {b}_0+{b}_1{N}_{\mathrm{H}3\mathrm{K}4\mathrm{me}3}+{b}_2{N}_{\mathrm{H}3\mathrm{K}27\mathrm{A}\mathrm{c}}+{b}_3{N}_{\mathrm{H}3\mathrm{K}27\mathrm{me}3}+{b}_4{N}_{\mathrm{H}3\mathrm{K}36\mathrm{me}3}+e $$

$$ \mathrm{Model}\ \mathrm{B}:\ \mathrm{mRNA}\ \mathrm{level} \sim {b}_0+{b}_1{N}_{\mathrm{H}3\mathrm{K}4\mathrm{me}3}+{b}_2{N}_{\mathrm{H}3\mathrm{K}27\mathrm{A}\mathrm{c}}+{b}_3{N}_{\mathrm{H}3\mathrm{K}27\mathrm{me}3}+{b}_4{N}_{\mathrm{H}3\mathrm{K}36\mathrm{me}3}+{b}_5\mathrm{half}\hbox{-} \mathrm{life}+e $$

Where *N* is studentized read coverage, mRNA level is log transformed RPKM, half-life is log transformed decay constant $\left(\lambda =\frac{ \log (2)}{\mathrm{half}\hbox{-} \mathrm{life}}\right)$ and *e* is the residual error.

### Analysis using ENCODE data

H3K4me3, H3K36me3, pol II and RNA-seq data for seven cell types were obtained from ENCODE and DLD-1 from DBTSS (see Additional file 1: Figure S5). Average enrichment for the H3K4me3 data ChIP-seq data was used to compare against the gene expression values.

### Western blot analysis

Cell lysates were prepared using RIPA buffer (50 mM Tris-Cl, pH 7.4, 150 mM NaCl, 5 mM EDTA, 1% Nonidet P-40, 1% sodium deoxycholate, 0.1% SDS, 1% proteinase inhibitor cocktail [Sigma-Aldrich, St. Louis, MO]). Proteins were resolved by 10% SDS PAGE and transferred to a polyvinylidene difluoride membrane. Membranes were incubated with the indicated primary antibodies, followed by incubation with anti-mouse or anti-rabbit secondary antibodies conjugated to horseradish peroxidase (HRP). After addition of the HRP substrate, the chemiluminescence signal was detected with a Luminescent Image Analyzer LAS-4000 (Fujifilm, Tokyo, Japan). Antibodies used for immunoblotting were as follows: rabbit anti-UPF1 (Abcam), rabbit anti-STAU1 (kindly provided by Dr. Ortín), rabbit anti-EXOSC5 antibody (Sigma-Aldrich, SAB200439), rabbit anti-actin (Sigma-Aldrich, A1978), and rabbit anti-tubulin (MBL, Nagoya, Japan).

### Availability of supporting data

Supporting sequence data are available through DDBJ under the accession number [DDBJ: DRA001215] and [DDBJ:DRA002961] and URL links to the sequencing data are available from http://trace.ddbj.nig.ac.jp/DRASearch/submission?acc=DRA001215 and http://trace.ddbj.nig.ac.jp/DRASearch/submission?acc=DRA002961.

## Additional files

Additional file 1: Figure S1. Statistics of the ChIP-seq, RNA-seq and BRIC-seq data used in the present study. a) The figure shows that the percentage of genes with high intensity H3K4me3 peaks increases as RPKM increases, whereas percentage of genes with low intensity H3K4me3 peaks or genes without peaks decrease as RPKM increases. b) ChIP-seq statistics. The number of peaks in ChIP-seq were called by MACS, irrespective of Refseq gene models. c) RNA-seq statistics. d) BRIC-seq statistics. **Figure S2.** ActD validation of BRIC-seq. **Figure S3.** Computational simulation on to the effect of siRNA knockdown to UPF1, EXOSC5 and STAU1. **Figure S4.** Expression level and RNA stability of HIC1 and ZNF783 transcription factors in indicated cells. **Figure S5.** List of ENCODE and DBTSS datasets used in this study. **Figure S6.** Scatterplots of the H3K4me3 intensities against gene expression values Y-axis indicates the H3K4me3 intensities and x-axis indicates gene expression. **Figure S7.** Number of ChIP (+)/RNA (−) genes in different cell types from ENCODE and DBTSS. **Figure S8.** Knockdown results for EXOSC5 and STAU1. **Figure S9.** List of siRNAs used for knockdown and oligonucleotides used for qPCR. **Figure S10.** Equations used in modeling the transcript levels. **Figure S11.** Statistics of the analysis conducted on ENCODE and DBTSS data. **Figure 12.** Boxplots show the nuclear to cytoplasm ratio of ENCODE and DBTSS data. Additional file 2: Table S1. Statistics into the correlation between ChIP-seq and RNA-seq. **Table S2.** RNAs potentially controlled by RNA turnover. **Table S3.** RNAs potentially controlled by UPF1, EXOSC5 and STAU1 as determined by data in the present study. **Table S4.** List of zinc figure proteins under the control of UPF1. **Table S5.** List of genes that may be controlled by RNA turnover from ENCODE data. **Table S6.** eRNAs coordinates that are potentially controlled by UPF1 (a) and EXOSC5 (b).

## Abbreviations

bp: Base pair
BRIC: 5′-bromouridine immunoprecipitation chase
BrU: 5′-bromouridine
ChIP: Chromatin immunoprecipitation
eRNA: Enhancer RNA
GO: Gene ontology
H3K4me1: Histone H3 mono-methylated lysine 4
H3K4me3: Histone H3 tri-methylated lysine 4
H3K27ac: Histone acetylated lysine 27
H3K36me3: Histone tri-methylated lysine 36
kbp: Kilobasepairs
lincRNA: Long intergenic non-coding
pol II: RNA polymerase II RNA
RPKM: Reads per kilobase per million mapped reads
