# Supplementary material for: Analysis of RNA decay factor mediated RNA stability contributions on RNA abundance
Source: BMC Genomics. 2015 Mar 6;16(1):154. doi: 10.1186/s12864-015-1358-y (PMC4359779; doi:10.1186/s12864-015-1358-y)
Supplement: Additional file 1: Figure S1. — Statistics of the ChIP-seq, RNA-seq and BRIC-seq data used in the present study. a) The figure shows that the percentage of genes with high intensity H3K4me3 peaks increases as RPKM increases, whereas percentage of genes with low intensity H3K4me3 peaks or genes without peaks decrease as RPKM increases. b) ChIP-seq statistics. The number of peaks in ChIP-seq were called by MACS, irrespective of Refseq gene models. c) RNA-seq statistics. d) BRIC-seq statistics. Figure S2. ActD validation of BRIC-seq. Figure S3. Computational simulation on to the effect of siRNA knockdown to UPF1, EXOSC5 and STAU1. Figure S4. Expression level and RNA stability of HIC1 and ZNF783 transcription factors in indicated cells. Figure S5. List of ENCODE and DBTSS datasets used in this study. Figure S6. Scatterplots of the H3K4me3 intensities against gene expression values Y-axis indicates the H3K4me3 intensities and x-axis indicates gene expression. Figure S7. Number of ChIP (+)/RNA (−) genes in different cell types from ENCODE and DBTSS. Figure S8. Knockdown results for EXOSC5 and STAU1. Figure S9. List of siRNAs used for knockdown and oligonucleotides used for qPCR. Figure S10. Equations used in modeling the transcript levels. Figure S11. Statistics of the analysis conducted on ENCODE and DBTSS data. Figure 12. Boxplots show the nuclear to cytoplasm ratio of ENCODE and DBTSS data. [file 12864_2015_1358_MOESM1_ESM.pptx]

## Slide 1
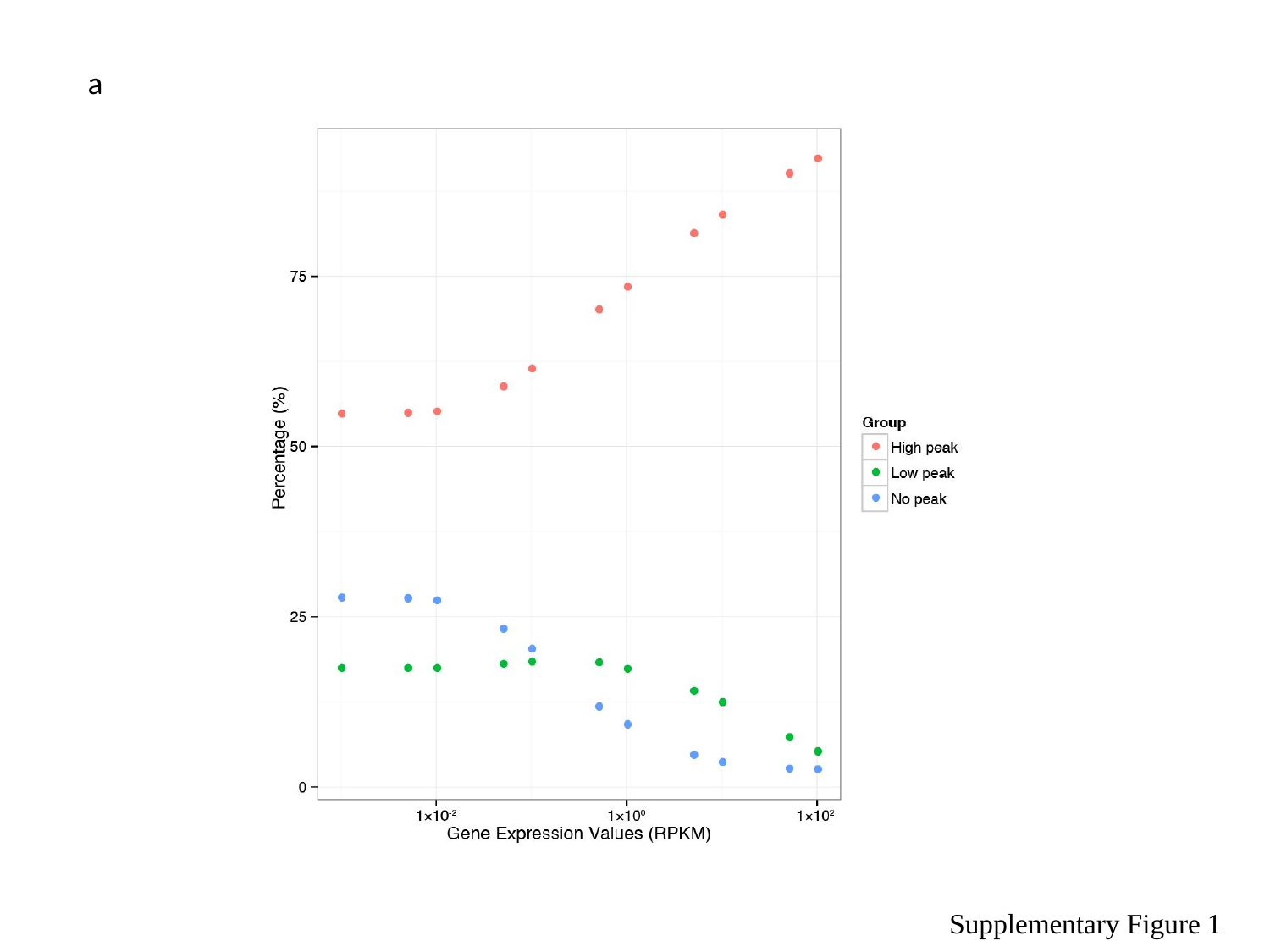

a
Supplementary Figure 1

## Slide 2
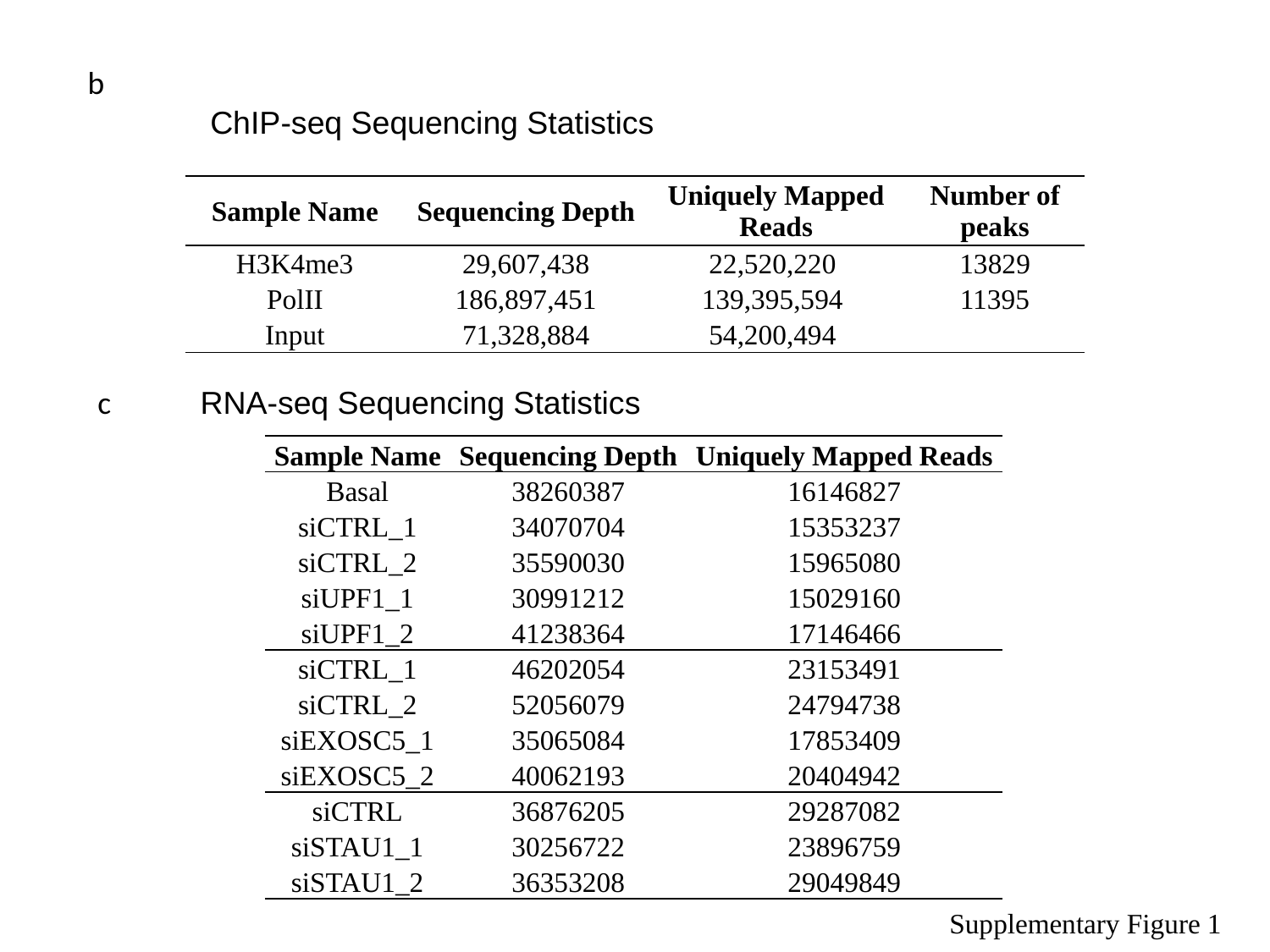

b
ChIP-seq Sequencing Statistics
| Sample Name | Sequencing Depth | Uniquely Mapped Reads | Number of peaks |
| --- | --- | --- | --- |
| H3K4me3 | 29,607,438 | 22,520,220 | 13829 |
| PolII | 186,897,451 | 139,395,594 | 11395 |
| Input | 71,328,884 | 54,200,494 | |
c
RNA-seq Sequencing Statistics
| Sample Name | Sequencing Depth | Uniquely Mapped Reads |
| --- | --- | --- |
| Basal | 38260387 | 16146827 |
| siCTRL\_1 | 34070704 | 15353237 |
| siCTRL\_2 | 35590030 | 15965080 |
| siUPF1\_1 | 30991212 | 15029160 |
| siUPF1\_2 | 41238364 | 17146466 |
| siCTRL\_1 | 46202054 | 23153491 |
| siCTRL\_2 | 52056079 | 24794738 |
| siEXOSC5\_1 | 35065084 | 17853409 |
| siEXOSC5\_2 | 40062193 | 20404942 |
| siCTRL | 36876205 | 29287082 |
| siSTAU1\_1 | 30256722 | 23896759 |
| siSTAU1\_2 | 36353208 | 29049849 |
Supplementary Figure 1

## Slide 3
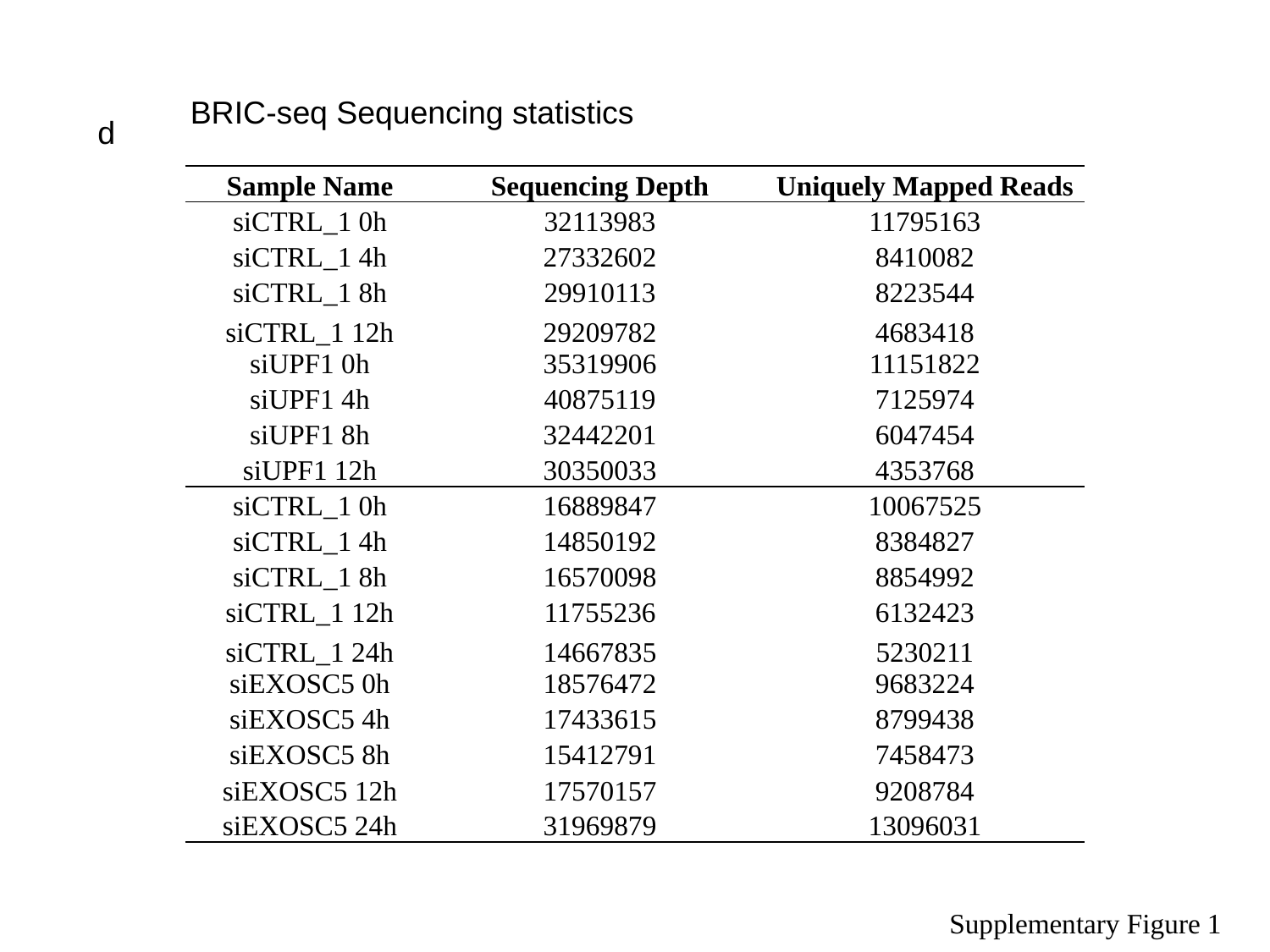

BRIC-seq Sequencing statistics
d
| Sample Name | Sequencing Depth | Uniquely Mapped Reads |
| --- | --- | --- |
| siCTRL\_1 0h | 32113983 | 11795163 |
| siCTRL\_1 4h | 27332602 | 8410082 |
| siCTRL\_1 8h | 29910113 | 8223544 |
| siCTRL\_1 12h siUPF1 0h | 29209782 35319906 | 4683418 11151822 |
| siUPF1 4h | 40875119 | 7125974 |
| siUPF1 8h | 32442201 | 6047454 |
| siUPF1 12h | 30350033 | 4353768 |
| siCTRL\_1 0h | 16889847 | 10067525 |
| siCTRL\_1 4h | 14850192 | 8384827 |
| siCTRL\_1 8h | 16570098 | 8854992 |
| siCTRL\_1 12h | 11755236 | 6132423 |
| siCTRL\_1 24h siEXOSC5 0h | 14667835 18576472 | 5230211 9683224 |
| siEXOSC5 4h | 17433615 | 8799438 |
| siEXOSC5 8h | 15412791 | 7458473 |
| siEXOSC5 12h | 17570157 | 9208784 |
| siEXOSC5 24h | 31969879 | 13096031 |
Supplementary Figure 1

## Slide 4
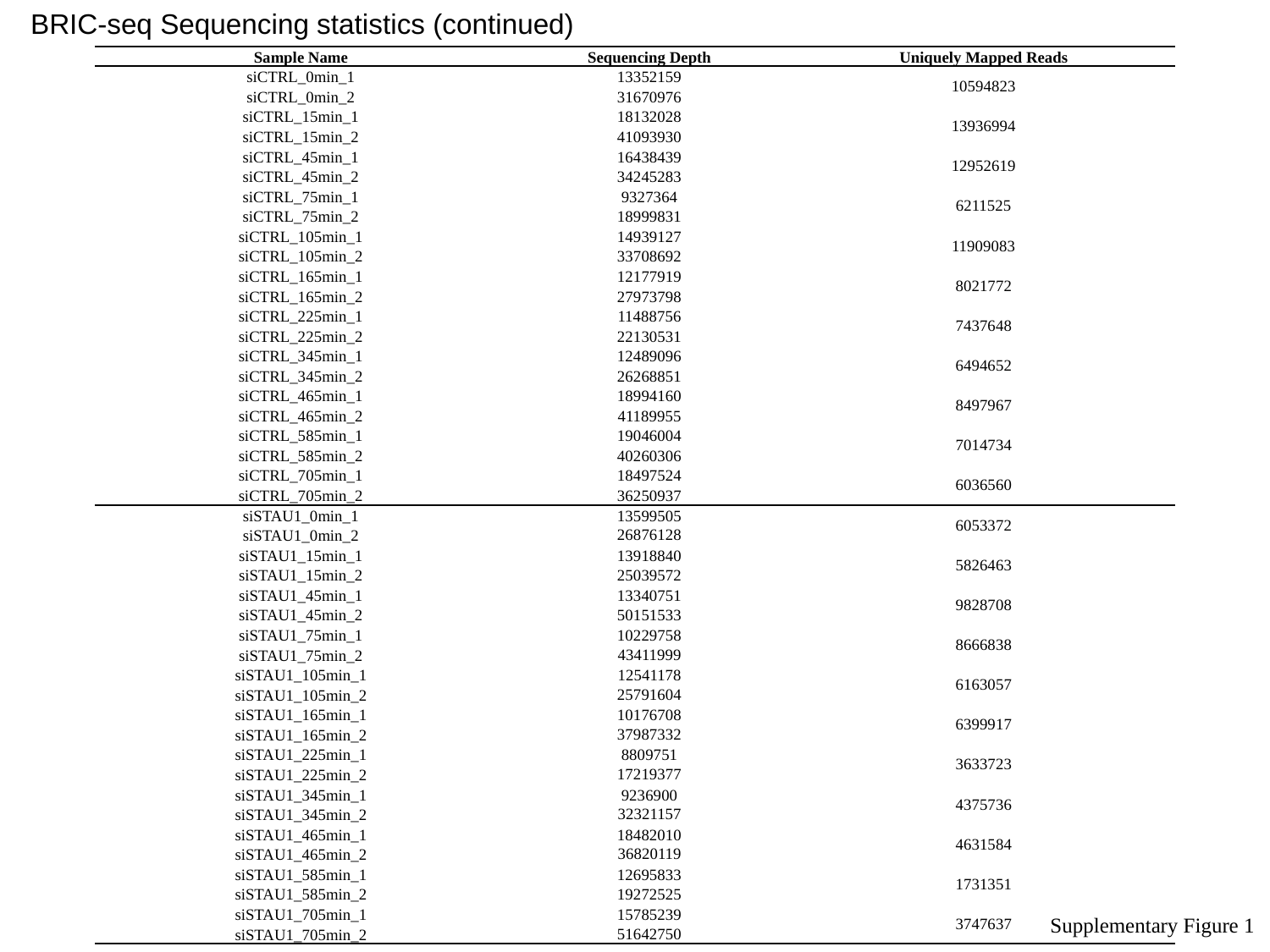

BRIC-seq Sequencing statistics (continued)
| Sample Name | Sequencing Depth | Uniquely Mapped Reads |
| --- | --- | --- |
| siCTRL\_0min\_1 | 13352159 | 10594823 |
| siCTRL\_0min\_2 | 31670976 | |
| siCTRL\_15min\_1 | 18132028 | 13936994 |
| siCTRL\_15min\_2 | 41093930 | |
| siCTRL\_45min\_1 | 16438439 | 12952619 |
| siCTRL\_45min\_2 | 34245283 | |
| siCTRL\_75min\_1 | 9327364 | 6211525 |
| siCTRL\_75min\_2 | 18999831 | |
| siCTRL\_105min\_1 | 14939127 | 11909083 |
| siCTRL\_105min\_2 | 33708692 | |
| siCTRL\_165min\_1 | 12177919 | 8021772 |
| siCTRL\_165min\_2 | 27973798 | |
| siCTRL\_225min\_1 | 11488756 | 7437648 |
| siCTRL\_225min\_2 | 22130531 | |
| siCTRL\_345min\_1 | 12489096 | 6494652 |
| siCTRL\_345min\_2 | 26268851 | |
| siCTRL\_465min\_1 | 18994160 | 8497967 |
| siCTRL\_465min\_2 | 41189955 | |
| siCTRL\_585min\_1 | 19046004 | 7014734 |
| siCTRL\_585min\_2 | 40260306 | |
| siCTRL\_705min\_1 | 18497524 | 6036560 |
| siCTRL\_705min\_2 | 36250937 | |
| siSTAU1\_0min\_1 | 13599505 | 6053372 |
| siSTAU1\_0min\_2 | 26876128 | |
| siSTAU1\_15min\_1 | 13918840 | 5826463 |
| siSTAU1\_15min\_2 | 25039572 | |
| siSTAU1\_45min\_1 | 13340751 | 9828708 |
| siSTAU1\_45min\_2 | 50151533 | |
| siSTAU1\_75min\_1 | 10229758 | 8666838 |
| siSTAU1\_75min\_2 | 43411999 | |
| siSTAU1\_105min\_1 | 12541178 | 6163057 |
| siSTAU1\_105min\_2 | 25791604 | |
| siSTAU1\_165min\_1 | 10176708 | 6399917 |
| siSTAU1\_165min\_2 | 37987332 | |
| siSTAU1\_225min\_1 | 8809751 | 3633723 |
| siSTAU1\_225min\_2 | 17219377 | |
| siSTAU1\_345min\_1 | 9236900 | 4375736 |
| siSTAU1\_345min\_2 | 32321157 | |
| siSTAU1\_465min\_1 | 18482010 | 4631584 |
| siSTAU1\_465min\_2 | 36820119 | |
| siSTAU1\_585min\_1 | 12695833 | 1731351 |
| siSTAU1\_585min\_2 | 19272525 | |
| siSTAU1\_705min\_1 | 15785239 | 3747637 |
| siSTAU1\_705min\_2 | 51642750 | |
Supplementary Figure 1

## Slide 5
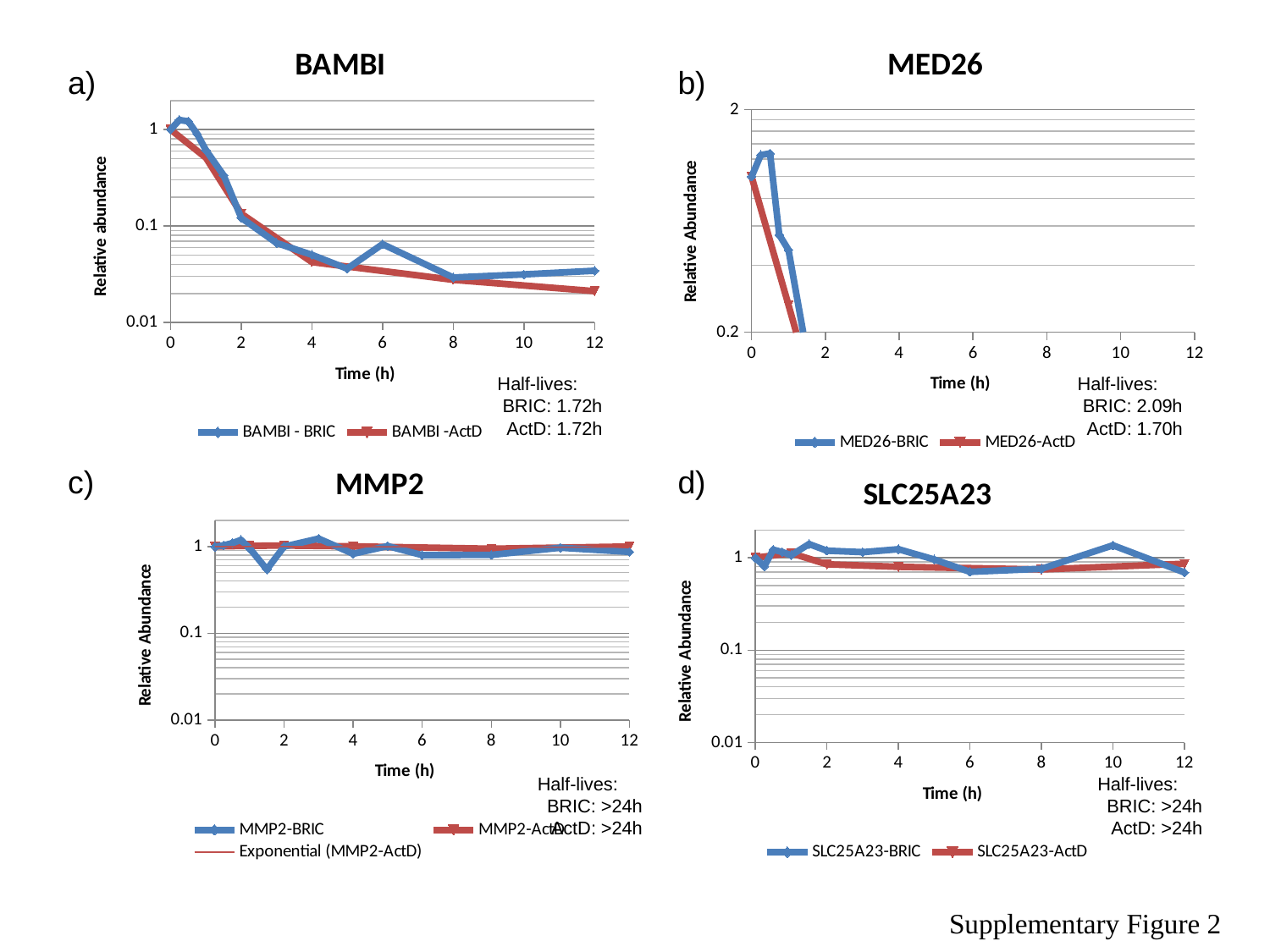

### Chart: BAMBI
| Category | BAMBI - BRIC | BAMBI -ActD |
|---|---|---|
### Chart: MED26
| Category | MED26-BRIC | MED26-ActD |
|---|---|---|a)
b)
Half-lives:
BRIC: 1.72h
ActD: 1.72h
Half-lives:
BRIC: 2.09h
ActD: 1.70h
### Chart: MMP2
| Category | MMP2-BRIC | MMP2-ActD |
|---|---|---|c)
### Chart: SLC25A23
| Category | SLC25A23-BRIC | SLC25A23-ActD |
|---|---|---|d)
Half-lives:
BRIC: >24h
ActD: >24h
Half-lives:
BRIC: >24h
ActD: >24h
Supplementary Figure 2

## Slide 6
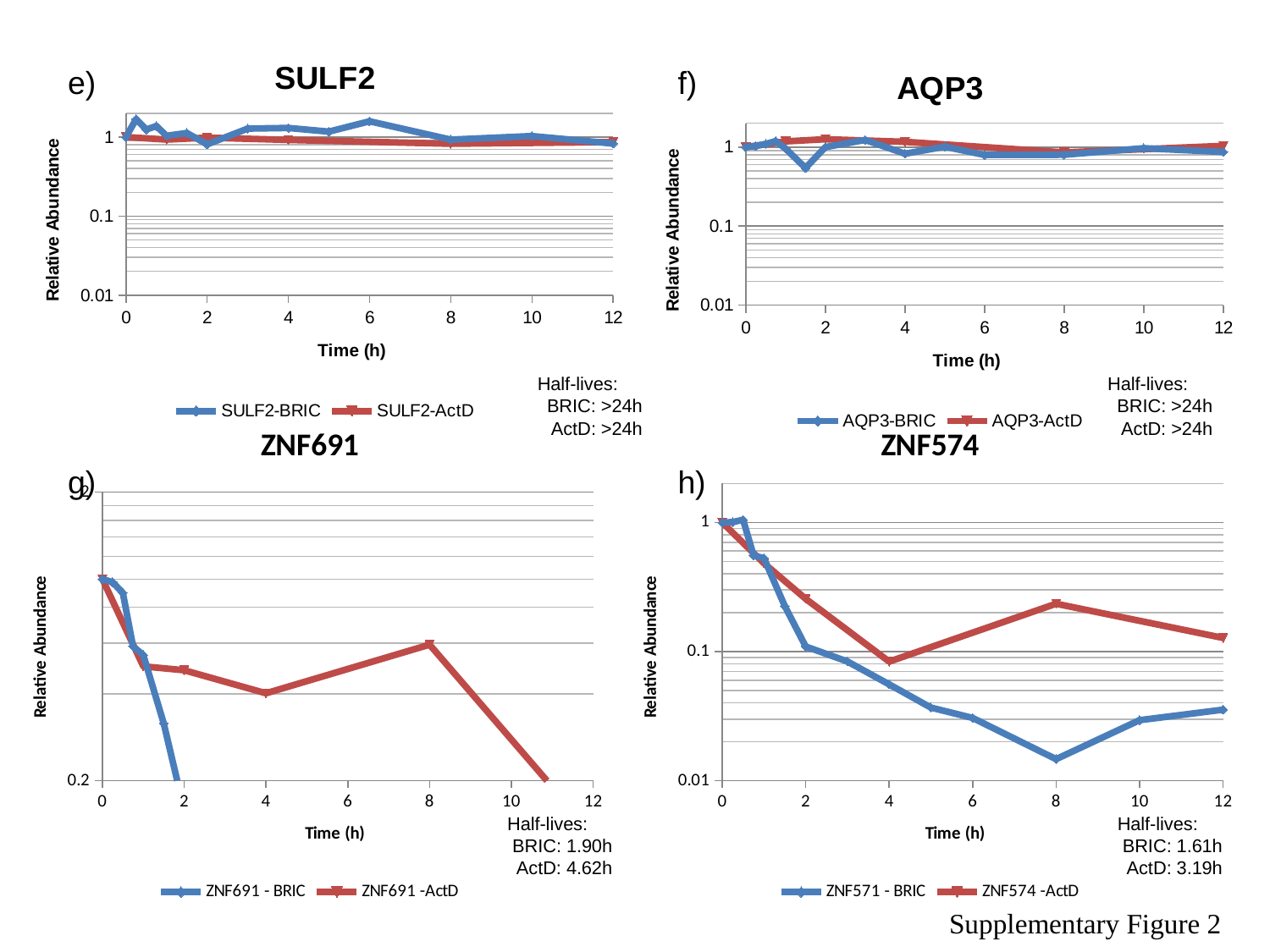

### Chart: SULF2
| Category | SULF2-BRIC | SULF2-ActD |
|---|---|---|
### Chart: AQP3
| Category | AQP3-BRIC | AQP3-ActD |
|---|---|---|e)
f)
Half-lives:
BRIC: >24h
ActD: >24h
Half-lives:
BRIC: >24h
ActD: >24h
### Chart: ZNF691
| Category | ZNF691 - BRIC | ZNF691 -ActD |
|---|---|---|
### Chart: ZNF574
| Category | ZNF571 - BRIC | ZNF574 -ActD |
|---|---|---|g)
h)
Half-lives:
BRIC: 1.90h
ActD: 4.62h
Half-lives:
BRIC: 1.61h
ActD: 3.19h
Supplementary Figure 2

## Slide 7
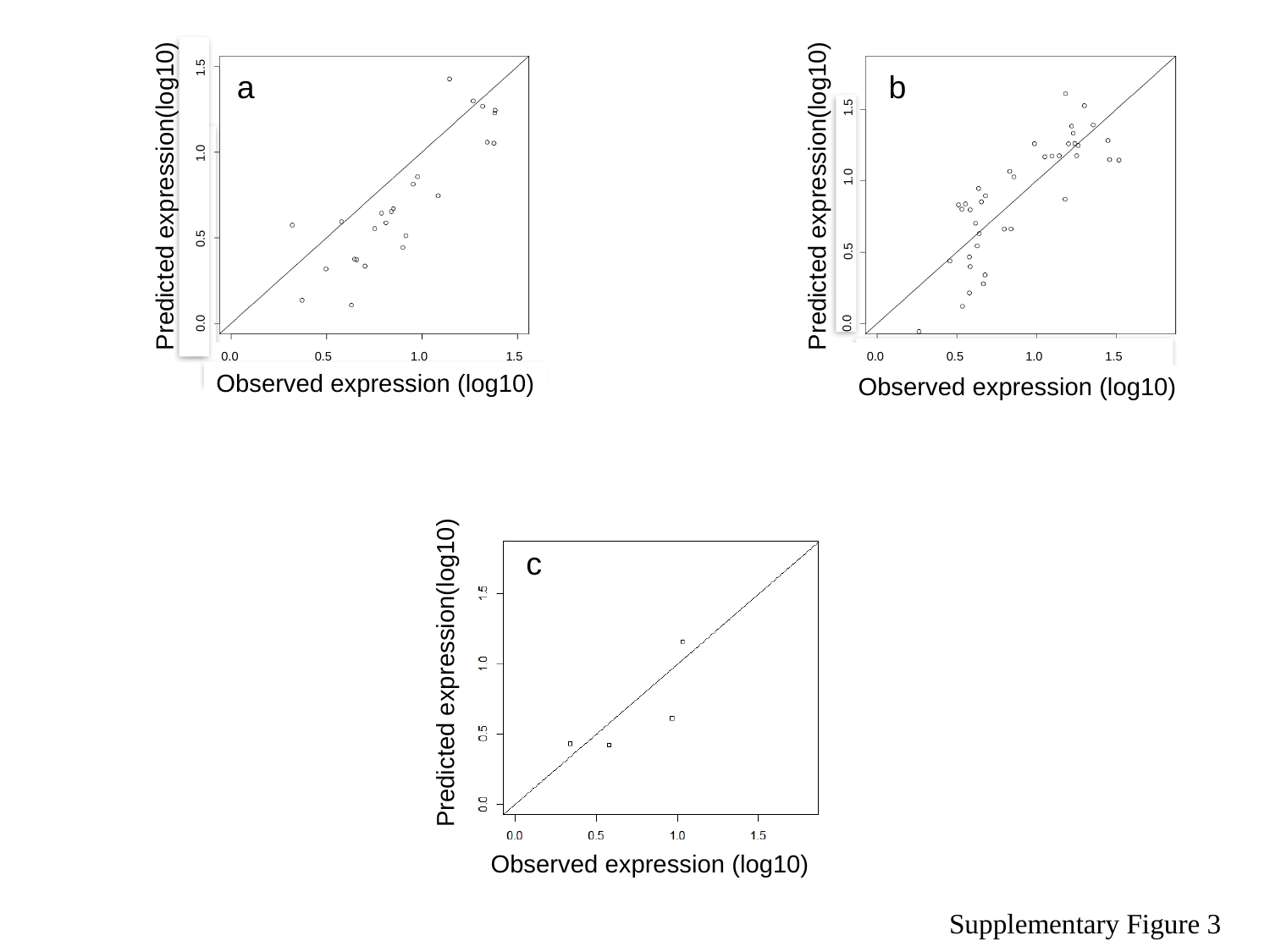

1.5
0.0
0.5
1.0
1.5
a
1.0
Predicted expression(log10)
0.5
0.0
Observed expression (log10)
b
1.5
1.0
Predicted expression(log10)
0.5
0.0
1.5
0.0
0.5
1.0
Observed expression (log10)
c
Predicted expression(log10)
Observed expression (log10)
Supplementary Figure 3

## Slide 8
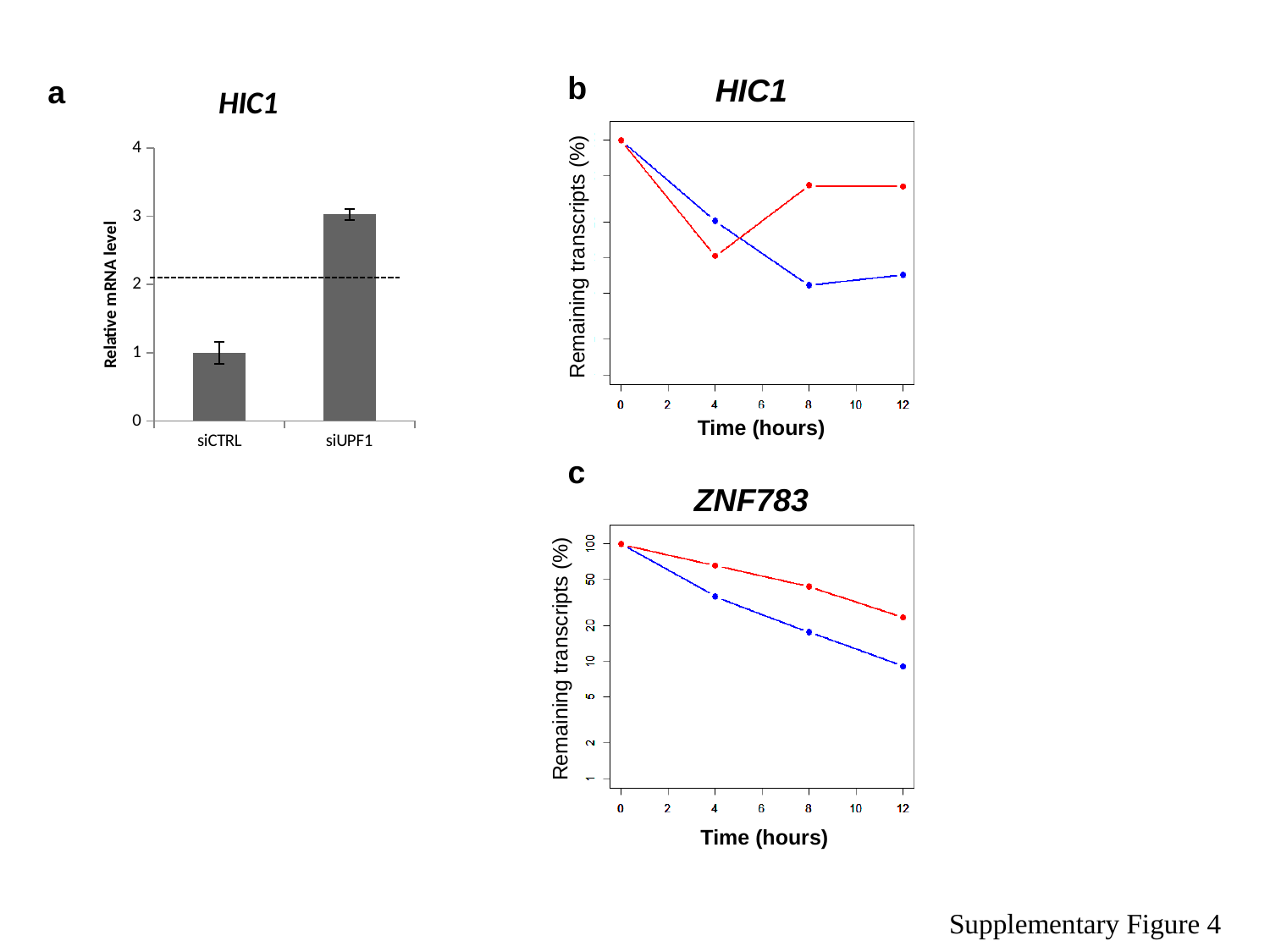

b
HIC1
a
### Chart: HIC1
| Category | |
|---|---|
| siCTRL | 1.0 |
| siUPF1 | 3.026308857 |Remaining transcripts (%)
Time (hours)
c
ZNF783
Remaining transcripts (%)
Time (hours)
Supplementary Figure 4

## Slide 9
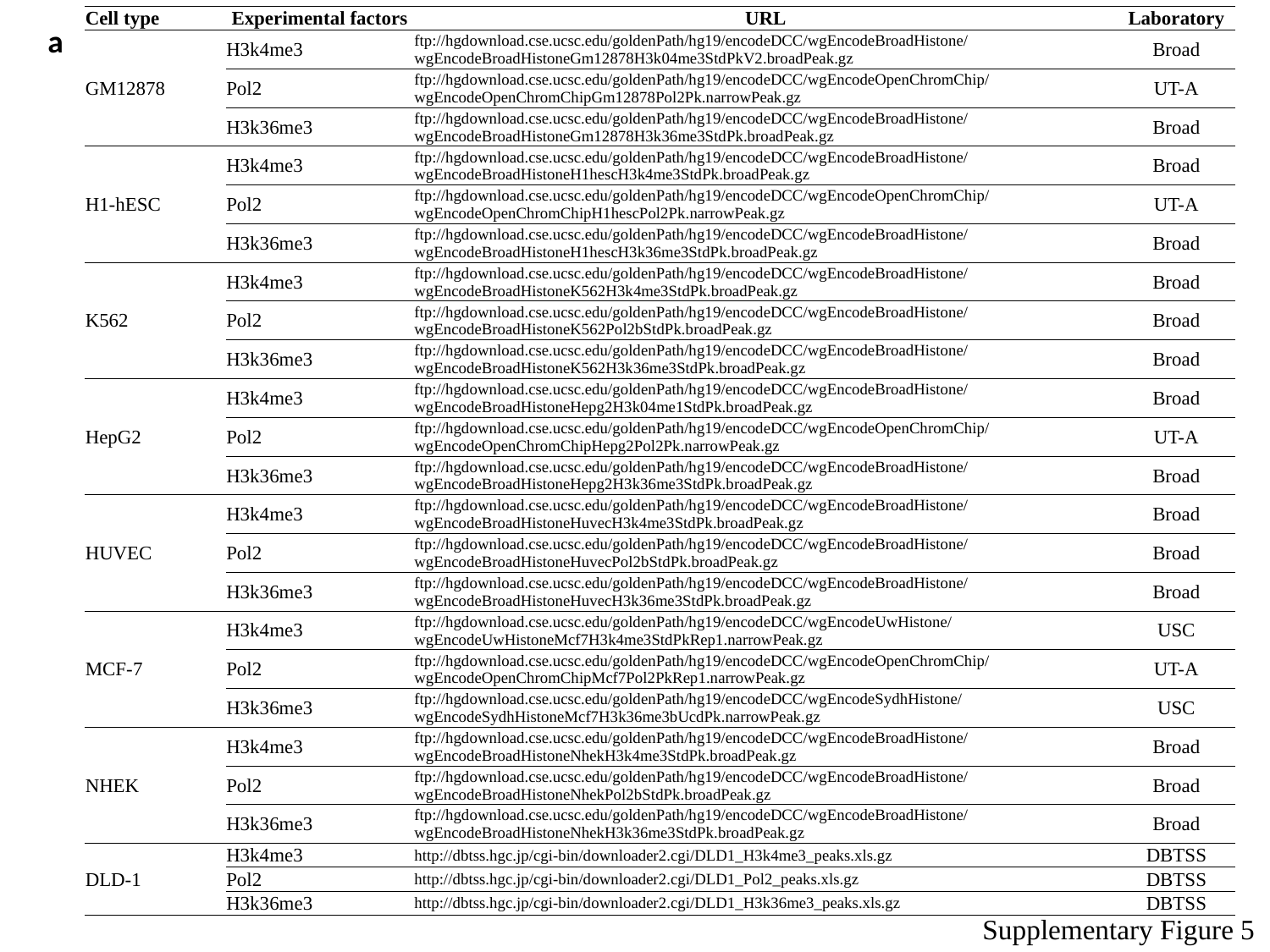

| Cell type | Experimental factors | URL | Laboratory |
| --- | --- | --- | --- |
| GM12878 | H3k4me3 | ftp://hgdownload.cse.ucsc.edu/goldenPath/hg19/encodeDCC/wgEncodeBroadHistone/wgEncodeBroadHistoneGm12878H3k04me3StdPkV2.broadPeak.gz | Broad |
| | Pol2 | ftp://hgdownload.cse.ucsc.edu/goldenPath/hg19/encodeDCC/wgEncodeOpenChromChip/wgEncodeOpenChromChipGm12878Pol2Pk.narrowPeak.gz | UT-A |
| | H3k36me3 | ftp://hgdownload.cse.ucsc.edu/goldenPath/hg19/encodeDCC/wgEncodeBroadHistone/wgEncodeBroadHistoneGm12878H3k36me3StdPk.broadPeak.gz | Broad |
| H1-hESC | H3k4me3 | ftp://hgdownload.cse.ucsc.edu/goldenPath/hg19/encodeDCC/wgEncodeBroadHistone/wgEncodeBroadHistoneH1hescH3k4me3StdPk.broadPeak.gz | Broad |
| | Pol2 | ftp://hgdownload.cse.ucsc.edu/goldenPath/hg19/encodeDCC/wgEncodeOpenChromChip/wgEncodeOpenChromChipH1hescPol2Pk.narrowPeak.gz | UT-A |
| | H3k36me3 | ftp://hgdownload.cse.ucsc.edu/goldenPath/hg19/encodeDCC/wgEncodeBroadHistone/wgEncodeBroadHistoneH1hescH3k36me3StdPk.broadPeak.gz | Broad |
| K562 | H3k4me3 | ftp://hgdownload.cse.ucsc.edu/goldenPath/hg19/encodeDCC/wgEncodeBroadHistone/wgEncodeBroadHistoneK562H3k4me3StdPk.broadPeak.gz | Broad |
| | Pol2 | ftp://hgdownload.cse.ucsc.edu/goldenPath/hg19/encodeDCC/wgEncodeBroadHistone/wgEncodeBroadHistoneK562Pol2bStdPk.broadPeak.gz | Broad |
| | H3k36me3 | ftp://hgdownload.cse.ucsc.edu/goldenPath/hg19/encodeDCC/wgEncodeBroadHistone/wgEncodeBroadHistoneK562H3k36me3StdPk.broadPeak.gz | Broad |
| HepG2 | H3k4me3 | ftp://hgdownload.cse.ucsc.edu/goldenPath/hg19/encodeDCC/wgEncodeBroadHistone/wgEncodeBroadHistoneHepg2H3k04me1StdPk.broadPeak.gz | Broad |
| | Pol2 | ftp://hgdownload.cse.ucsc.edu/goldenPath/hg19/encodeDCC/wgEncodeOpenChromChip/wgEncodeOpenChromChipHepg2Pol2Pk.narrowPeak.gz | UT-A |
| | H3k36me3 | ftp://hgdownload.cse.ucsc.edu/goldenPath/hg19/encodeDCC/wgEncodeBroadHistone/wgEncodeBroadHistoneHepg2H3k36me3StdPk.broadPeak.gz | Broad |
| HUVEC | H3k4me3 | ftp://hgdownload.cse.ucsc.edu/goldenPath/hg19/encodeDCC/wgEncodeBroadHistone/wgEncodeBroadHistoneHuvecH3k4me3StdPk.broadPeak.gz | Broad |
| | Pol2 | ftp://hgdownload.cse.ucsc.edu/goldenPath/hg19/encodeDCC/wgEncodeBroadHistone/wgEncodeBroadHistoneHuvecPol2bStdPk.broadPeak.gz | Broad |
| | H3k36me3 | ftp://hgdownload.cse.ucsc.edu/goldenPath/hg19/encodeDCC/wgEncodeBroadHistone/wgEncodeBroadHistoneHuvecH3k36me3StdPk.broadPeak.gz | Broad |
| MCF-7 | H3k4me3 | ftp://hgdownload.cse.ucsc.edu/goldenPath/hg19/encodeDCC/wgEncodeUwHistone/wgEncodeUwHistoneMcf7H3k4me3StdPkRep1.narrowPeak.gz | USC |
| | Pol2 | ftp://hgdownload.cse.ucsc.edu/goldenPath/hg19/encodeDCC/wgEncodeOpenChromChip/wgEncodeOpenChromChipMcf7Pol2PkRep1.narrowPeak.gz | UT-A |
| | H3k36me3 | ftp://hgdownload.cse.ucsc.edu/goldenPath/hg19/encodeDCC/wgEncodeSydhHistone/wgEncodeSydhHistoneMcf7H3k36me3bUcdPk.narrowPeak.gz | USC |
| NHEK | H3k4me3 | ftp://hgdownload.cse.ucsc.edu/goldenPath/hg19/encodeDCC/wgEncodeBroadHistone/wgEncodeBroadHistoneNhekH3k4me3StdPk.broadPeak.gz | Broad |
| | Pol2 | ftp://hgdownload.cse.ucsc.edu/goldenPath/hg19/encodeDCC/wgEncodeBroadHistone/wgEncodeBroadHistoneNhekPol2bStdPk.broadPeak.gz | Broad |
| | H3k36me3 | ftp://hgdownload.cse.ucsc.edu/goldenPath/hg19/encodeDCC/wgEncodeBroadHistone/wgEncodeBroadHistoneNhekH3k36me3StdPk.broadPeak.gz | Broad |
| DLD-1 | H3k4me3 | http://dbtss.hgc.jp/cgi-bin/downloader2.cgi/DLD1\_H3k4me3\_peaks.xls.gz | DBTSS |
| | Pol2 | http://dbtss.hgc.jp/cgi-bin/downloader2.cgi/DLD1\_Pol2\_peaks.xls.gz | DBTSS |
| | H3k36me3 | http://dbtss.hgc.jp/cgi-bin/downloader2.cgi/DLD1\_H3k36me3\_peaks.xls.gz | DBTSS |
a
Supplementary Figure 5

## Slide 10
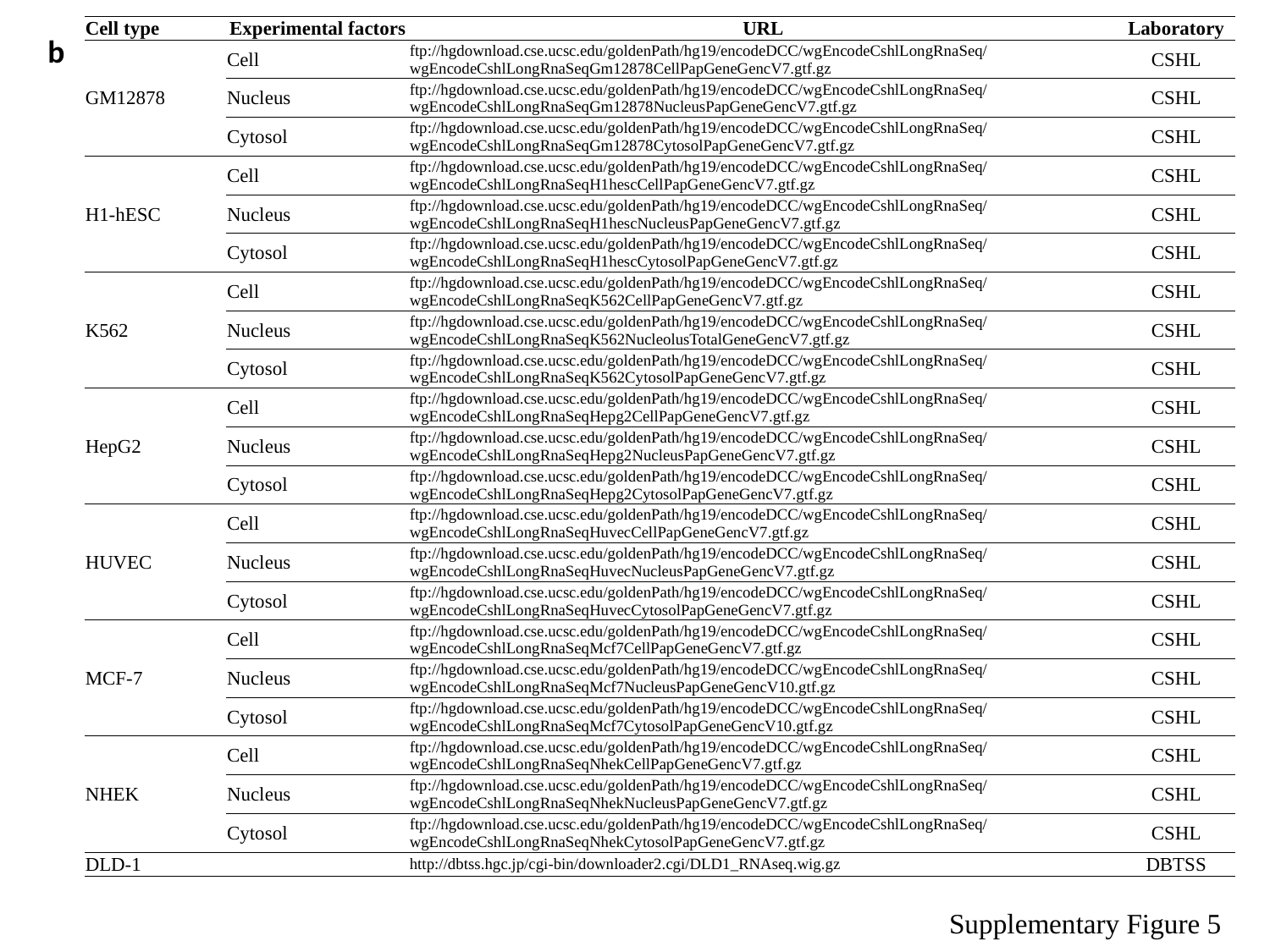

| Cell type | Experimental factors | URL | Laboratory |
| --- | --- | --- | --- |
| GM12878 | Cell | ftp://hgdownload.cse.ucsc.edu/goldenPath/hg19/encodeDCC/wgEncodeCshlLongRnaSeq/wgEncodeCshlLongRnaSeqGm12878CellPapGeneGencV7.gtf.gz | CSHL |
| | Nucleus | ftp://hgdownload.cse.ucsc.edu/goldenPath/hg19/encodeDCC/wgEncodeCshlLongRnaSeq/wgEncodeCshlLongRnaSeqGm12878NucleusPapGeneGencV7.gtf.gz | CSHL |
| | Cytosol | ftp://hgdownload.cse.ucsc.edu/goldenPath/hg19/encodeDCC/wgEncodeCshlLongRnaSeq/wgEncodeCshlLongRnaSeqGm12878CytosolPapGeneGencV7.gtf.gz | CSHL |
| H1-hESC | Cell | ftp://hgdownload.cse.ucsc.edu/goldenPath/hg19/encodeDCC/wgEncodeCshlLongRnaSeq/wgEncodeCshlLongRnaSeqH1hescCellPapGeneGencV7.gtf.gz | CSHL |
| | Nucleus | ftp://hgdownload.cse.ucsc.edu/goldenPath/hg19/encodeDCC/wgEncodeCshlLongRnaSeq/wgEncodeCshlLongRnaSeqH1hescNucleusPapGeneGencV7.gtf.gz | CSHL |
| | Cytosol | ftp://hgdownload.cse.ucsc.edu/goldenPath/hg19/encodeDCC/wgEncodeCshlLongRnaSeq/wgEncodeCshlLongRnaSeqH1hescCytosolPapGeneGencV7.gtf.gz | CSHL |
| K562 | Cell | ftp://hgdownload.cse.ucsc.edu/goldenPath/hg19/encodeDCC/wgEncodeCshlLongRnaSeq/wgEncodeCshlLongRnaSeqK562CellPapGeneGencV7.gtf.gz | CSHL |
| | Nucleus | ftp://hgdownload.cse.ucsc.edu/goldenPath/hg19/encodeDCC/wgEncodeCshlLongRnaSeq/wgEncodeCshlLongRnaSeqK562NucleolusTotalGeneGencV7.gtf.gz | CSHL |
| | Cytosol | ftp://hgdownload.cse.ucsc.edu/goldenPath/hg19/encodeDCC/wgEncodeCshlLongRnaSeq/wgEncodeCshlLongRnaSeqK562CytosolPapGeneGencV7.gtf.gz | CSHL |
| HepG2 | Cell | ftp://hgdownload.cse.ucsc.edu/goldenPath/hg19/encodeDCC/wgEncodeCshlLongRnaSeq/wgEncodeCshlLongRnaSeqHepg2CellPapGeneGencV7.gtf.gz | CSHL |
| | Nucleus | ftp://hgdownload.cse.ucsc.edu/goldenPath/hg19/encodeDCC/wgEncodeCshlLongRnaSeq/wgEncodeCshlLongRnaSeqHepg2NucleusPapGeneGencV7.gtf.gz | CSHL |
| | Cytosol | ftp://hgdownload.cse.ucsc.edu/goldenPath/hg19/encodeDCC/wgEncodeCshlLongRnaSeq/wgEncodeCshlLongRnaSeqHepg2CytosolPapGeneGencV7.gtf.gz | CSHL |
| HUVEC | Cell | ftp://hgdownload.cse.ucsc.edu/goldenPath/hg19/encodeDCC/wgEncodeCshlLongRnaSeq/wgEncodeCshlLongRnaSeqHuvecCellPapGeneGencV7.gtf.gz | CSHL |
| | Nucleus | ftp://hgdownload.cse.ucsc.edu/goldenPath/hg19/encodeDCC/wgEncodeCshlLongRnaSeq/wgEncodeCshlLongRnaSeqHuvecNucleusPapGeneGencV7.gtf.gz | CSHL |
| | Cytosol | ftp://hgdownload.cse.ucsc.edu/goldenPath/hg19/encodeDCC/wgEncodeCshlLongRnaSeq/wgEncodeCshlLongRnaSeqHuvecCytosolPapGeneGencV7.gtf.gz | CSHL |
| MCF-7 | Cell | ftp://hgdownload.cse.ucsc.edu/goldenPath/hg19/encodeDCC/wgEncodeCshlLongRnaSeq/wgEncodeCshlLongRnaSeqMcf7CellPapGeneGencV7.gtf.gz | CSHL |
| | Nucleus | ftp://hgdownload.cse.ucsc.edu/goldenPath/hg19/encodeDCC/wgEncodeCshlLongRnaSeq/wgEncodeCshlLongRnaSeqMcf7NucleusPapGeneGencV10.gtf.gz | CSHL |
| | Cytosol | ftp://hgdownload.cse.ucsc.edu/goldenPath/hg19/encodeDCC/wgEncodeCshlLongRnaSeq/wgEncodeCshlLongRnaSeqMcf7CytosolPapGeneGencV10.gtf.gz | CSHL |
| NHEK | Cell | ftp://hgdownload.cse.ucsc.edu/goldenPath/hg19/encodeDCC/wgEncodeCshlLongRnaSeq/wgEncodeCshlLongRnaSeqNhekCellPapGeneGencV7.gtf.gz | CSHL |
| | Nucleus | ftp://hgdownload.cse.ucsc.edu/goldenPath/hg19/encodeDCC/wgEncodeCshlLongRnaSeq/wgEncodeCshlLongRnaSeqNhekNucleusPapGeneGencV7.gtf.gz | CSHL |
| | Cytosol | ftp://hgdownload.cse.ucsc.edu/goldenPath/hg19/encodeDCC/wgEncodeCshlLongRnaSeq/wgEncodeCshlLongRnaSeqNhekCytosolPapGeneGencV7.gtf.gz | CSHL |
| DLD-1 | | http://dbtss.hgc.jp/cgi-bin/downloader2.cgi/DLD1\_RNAseq.wig.gz | DBTSS |
b
Supplementary Figure 5

## Slide 11
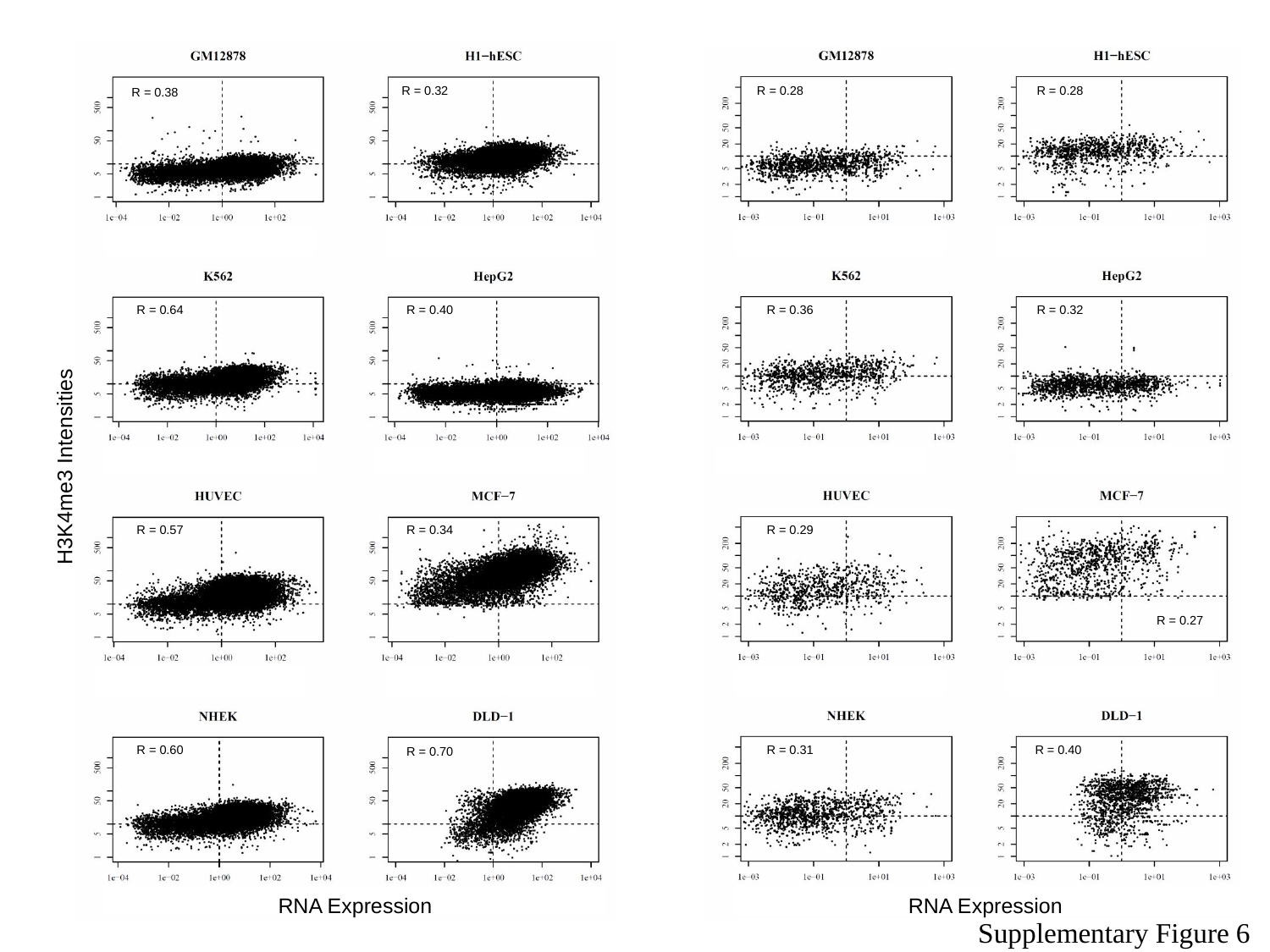

R = 0.32
R = 0.28
R = 0.28
R = 0.38
R = 0.64
R = 0.40
R = 0.36
R = 0.32
H3K4me3 Intensities
R = 0.57
R = 0.34
R = 0.29
R = 0.27
R = 0.60
R = 0.31
R = 0.40
R = 0.70
RNA Expression
RNA Expression
Supplementary Figure 6

## Slide 12
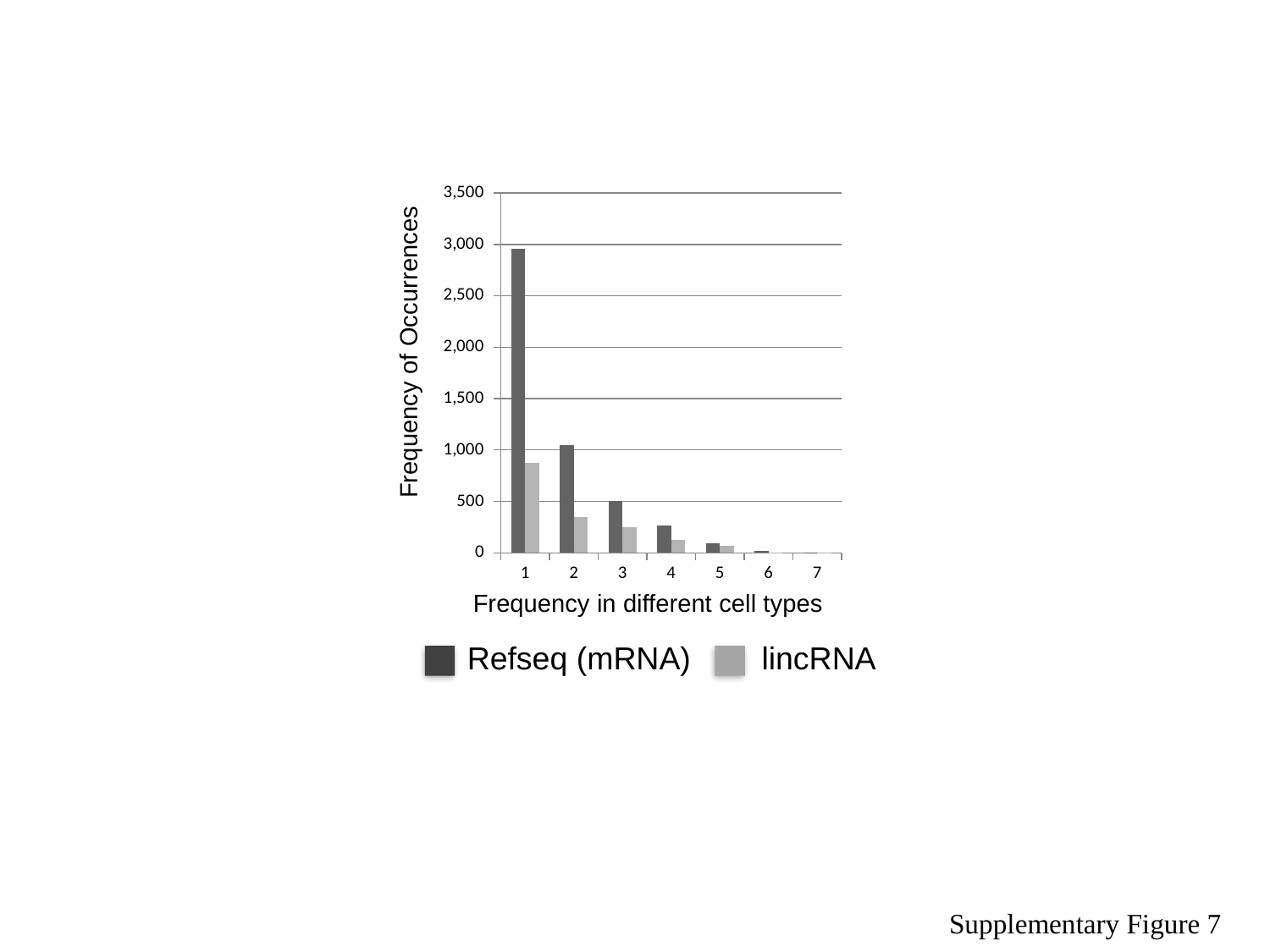

### Chart
| Category | Refseq | lincRNA |
|---|---|---|Frequency of Occurrences
Frequency in different cell types
Refseq (mRNA) lincRNA
Supplementary Figure 7

## Slide 13
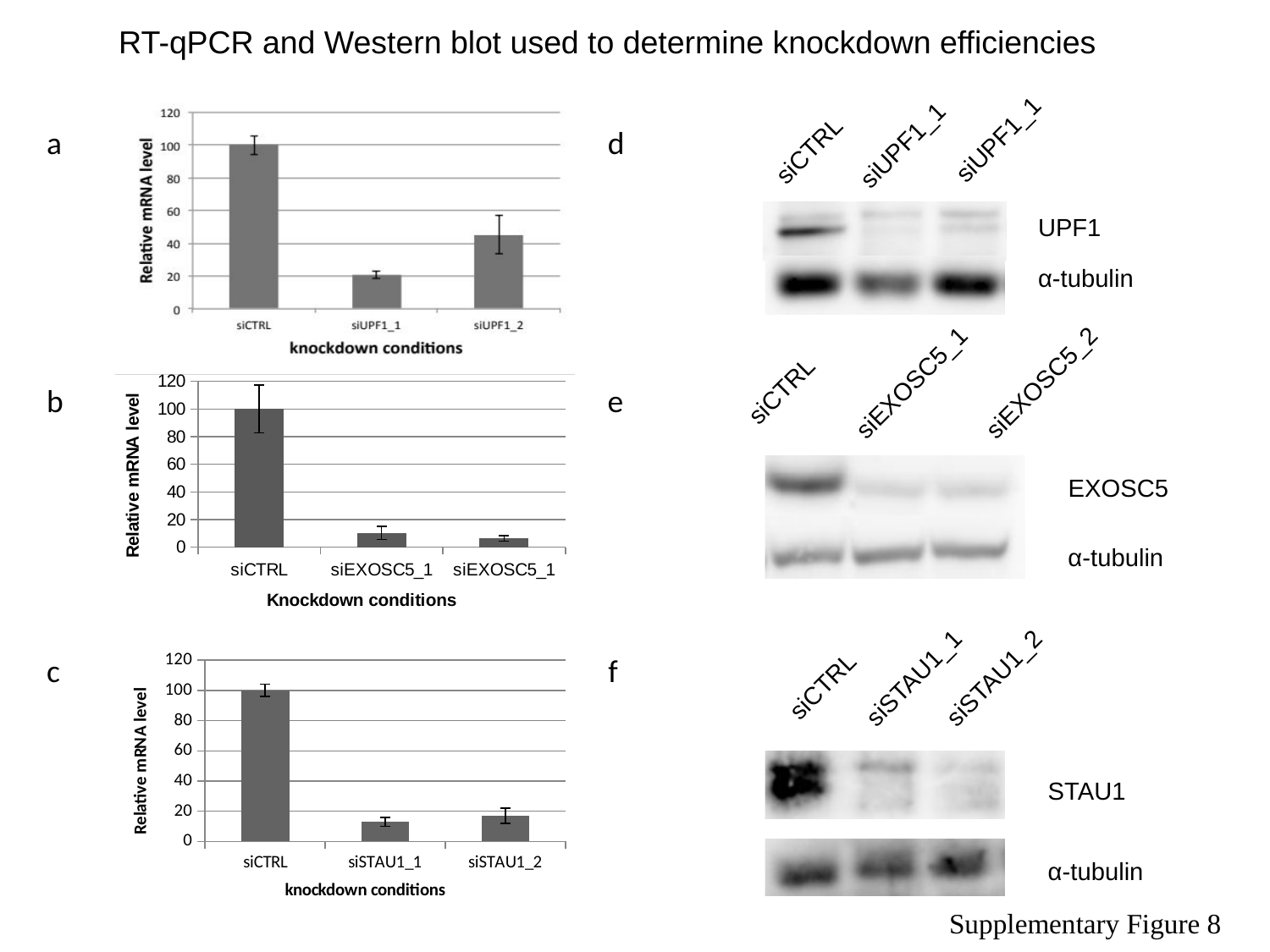

RT-qPCR and Western blot used to determine knockdown efficiencies
a
d
siUPF1_1
siUPF1_1
siCTRL
UPF1
α-tubulin
siEXOSC5_1
siEXOSC5_2
### Chart
| Category | |
|---|---|
| siCTRL | 100.0 |
| siEXOSC5_1 | 10.2 |
| siEXOSC5_1 | 6.3 |siCTRL
b
e
EXOSC5
α-tubulin
c
f
### Chart
| Category | |
|---|---|
| siCTRL | 100.0 |
| siSTAU1_1 | 13.0 |
| siSTAU1_2 | 17.0 |siSTAU1_1
siSTAU1_2
siCTRL
STAU1
α-tubulin
Supplementary Figure 8

## Slide 14
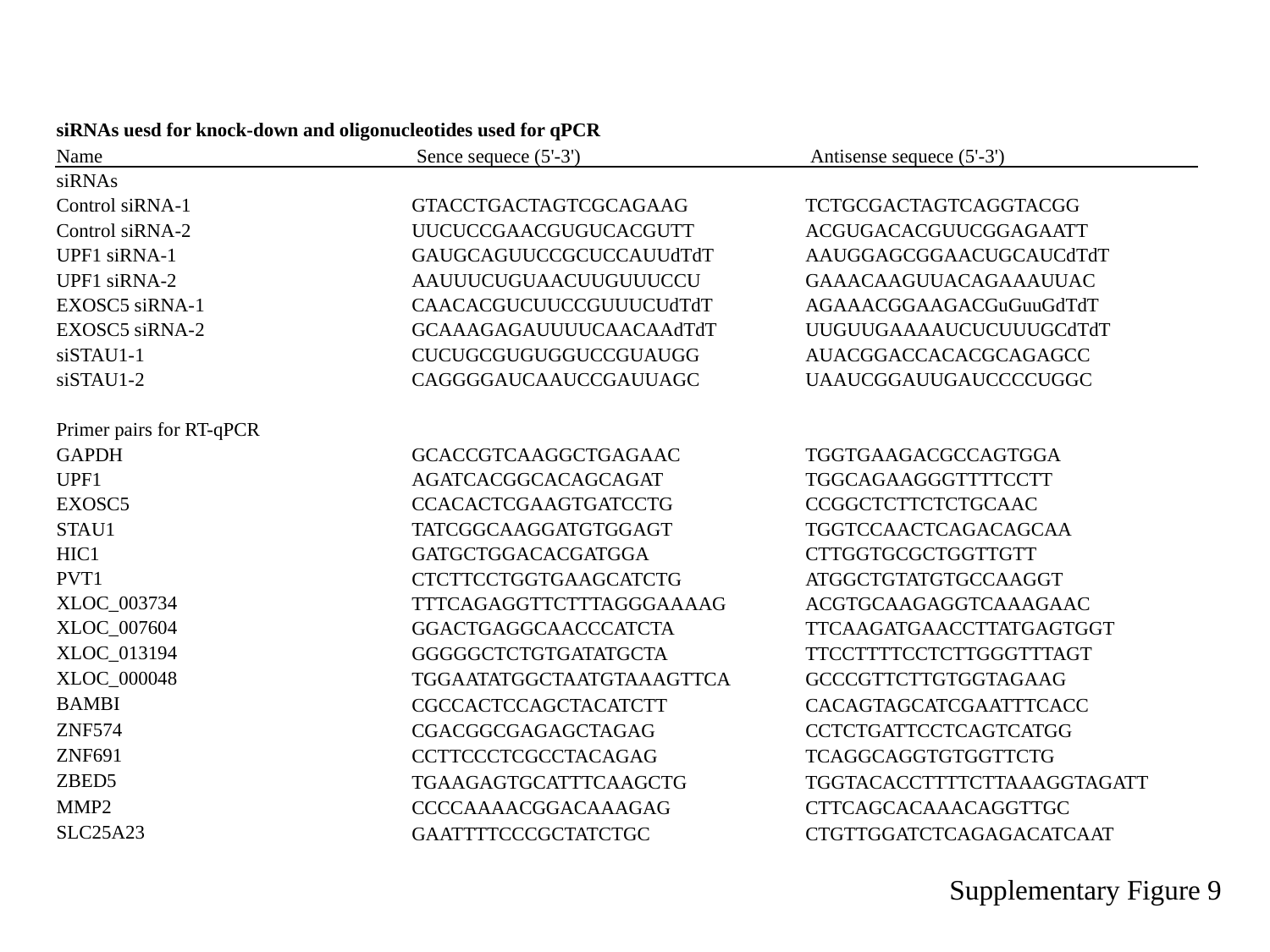

| siRNAs uesd for knock-down and oligonucleotides used for qPCR | | |
| --- | --- | --- |
| Name | Sence sequece (5'-3') | Antisense sequece (5'-3') |
| siRNAs | | |
| Control siRNA-1 | GTACCTGACTAGTCGCAGAAG | TCTGCGACTAGTCAGGTACGG |
| Control siRNA-2 | UUCUCCGAACGUGUCACGUTT | ACGUGACACGUUCGGAGAATT |
| UPF1 siRNA-1 | GAUGCAGUUCCGCUCCAUUdTdT | AAUGGAGCGGAACUGCAUCdTdT |
| UPF1 siRNA-2 | AAUUUCUGUAACUUGUUUCCU | GAAACAAGUUACAGAAAUUAC |
| EXOSC5 siRNA-1 | CAACACGUCUUCCGUUUCUdTdT | AGAAACGGAAGACGuGuuGdTdT |
| EXOSC5 siRNA-2 | GCAAAGAGAUUUUCAACAAdTdT | UUGUUGAAAAUCUCUUUGCdTdT |
| siSTAU1-1 | CUCUGCGUGUGGUCCGUAUGG | AUACGGACCACACGCAGAGCC |
| siSTAU1-2 | CAGGGGAUCAAUCCGAUUAGC | UAAUCGGAUUGAUCCCCUGGC |
| | | |
| Primer pairs for RT-qPCR | | |
| GAPDH | GCACCGTCAAGGCTGAGAAC | TGGTGAAGACGCCAGTGGA |
| UPF1 | AGATCACGGCACAGCAGAT | TGGCAGAAGGGTTTTCCTT |
| EXOSC5 | CCACACTCGAAGTGATCCTG | CCGGCTCTTCTCTGCAAC |
| STAU1 | TATCGGCAAGGATGTGGAGT | TGGTCCAACTCAGACAGCAA |
| HIC1 | GATGCTGGACACGATGGA | CTTGGTGCGCTGGTTGTT |
| PVT1 | CTCTTCCTGGTGAAGCATCTG | ATGGCTGTATGTGCCAAGGT |
| XLOC\_003734 | TTTCAGAGGTTCTTTAGGGAAAAG | ACGTGCAAGAGGTCAAAGAAC |
| XLOC\_007604 | GGACTGAGGCAACCCATCTA | TTCAAGATGAACCTTATGAGTGGT |
| XLOC\_013194 | GGGGGCTCTGTGATATGCTA | TTCCTTTTCCTCTTGGGTTTAGT |
| XLOC\_000048 | TGGAATATGGCTAATGTAAAGTTCA | GCCCGTTCTTGTGGTAGAAG |
| BAMBI | CGCCACTCCAGCTACATCTT | CACAGTAGCATCGAATTTCACC |
| ZNF574 | CGACGGCGAGAGCTAGAG | CCTCTGATTCCTCAGTCATGG |
| ZNF691 | CCTTCCCTCGCCTACAGAG | TCAGGCAGGTGTGGTTCTG |
| ZBED5 | TGAAGAGTGCATTTCAAGCTG | TGGTACACCTTTTCTTAAAGGTAGATT |
| MMP2 | CCCCAAAACGGACAAAGAG | CTTCAGCACAAACAGGTTGC |
| SLC25A23 | GAATTTTCCCGCTATCTGC | CTGTTGGATCTCAGAGACATCAAT |
Supplementary Figure 9

## Slide 15
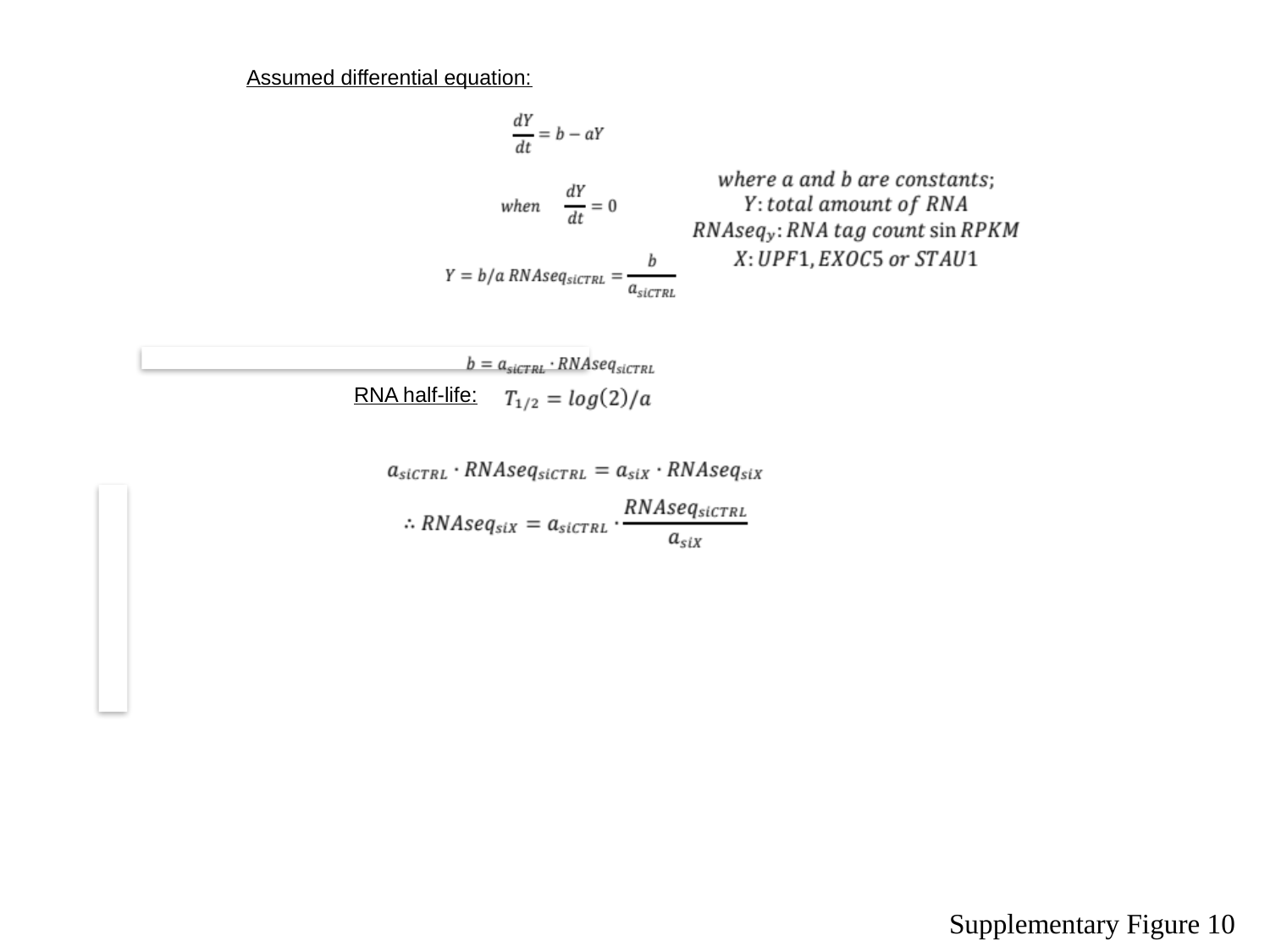

Assumed differential equation:
RNA half-life:
Supplementary Figure 10

## Slide 16
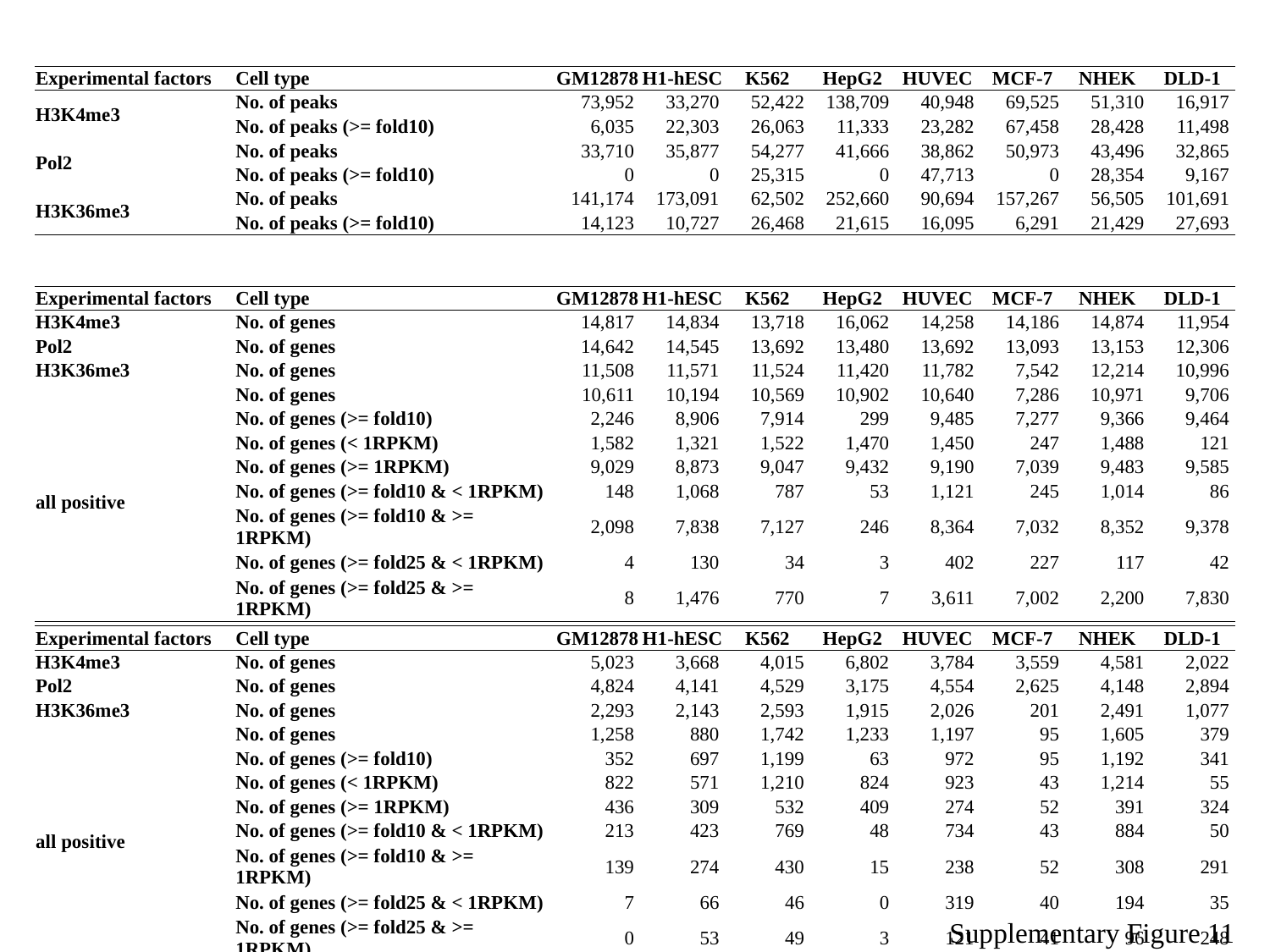

| Experimental factors | Cell type | GM12878 | H1-hESC | K562 | HepG2 | HUVEC | MCF-7 | NHEK | DLD-1 |
| --- | --- | --- | --- | --- | --- | --- | --- | --- | --- |
| H3K4me3 | No. of peaks | 73,952 | 33,270 | 52,422 | 138,709 | 40,948 | 69,525 | 51,310 | 16,917 |
| | No. of peaks (>= fold10) | 6,035 | 22,303 | 26,063 | 11,333 | 23,282 | 67,458 | 28,428 | 11,498 |
| Pol2 | No. of peaks | 33,710 | 35,877 | 54,277 | 41,666 | 38,862 | 50,973 | 43,496 | 32,865 |
| | No. of peaks (>= fold10) | 0 | 0 | 25,315 | 0 | 47,713 | 0 | 28,354 | 9,167 |
| H3K36me3 | No. of peaks | 141,174 | 173,091 | 62,502 | 252,660 | 90,694 | 157,267 | 56,505 | 101,691 |
| | No. of peaks (>= fold10) | 14,123 | 10,727 | 26,468 | 21,615 | 16,095 | 6,291 | 21,429 | 27,693 |
| Experimental factors | Cell type | GM12878 | H1-hESC | K562 | HepG2 | HUVEC | MCF-7 | NHEK | DLD-1 |
| --- | --- | --- | --- | --- | --- | --- | --- | --- | --- |
| H3K4me3 | No. of genes | 14,817 | 14,834 | 13,718 | 16,062 | 14,258 | 14,186 | 14,874 | 11,954 |
| Pol2 | No. of genes | 14,642 | 14,545 | 13,692 | 13,480 | 13,692 | 13,093 | 13,153 | 12,306 |
| H3K36me3 | No. of genes | 11,508 | 11,571 | 11,524 | 11,420 | 11,782 | 7,542 | 12,214 | 10,996 |
| all positive | No. of genes | 10,611 | 10,194 | 10,569 | 10,902 | 10,640 | 7,286 | 10,971 | 9,706 |
| | No. of genes (>= fold10) | 2,246 | 8,906 | 7,914 | 299 | 9,485 | 7,277 | 9,366 | 9,464 |
| | No. of genes (< 1RPKM) | 1,582 | 1,321 | 1,522 | 1,470 | 1,450 | 247 | 1,488 | 121 |
| | No. of genes (>= 1RPKM) | 9,029 | 8,873 | 9,047 | 9,432 | 9,190 | 7,039 | 9,483 | 9,585 |
| | No. of genes (>= fold10 & < 1RPKM) | 148 | 1,068 | 787 | 53 | 1,121 | 245 | 1,014 | 86 |
| | No. of genes (>= fold10 & >= 1RPKM) | 2,098 | 7,838 | 7,127 | 246 | 8,364 | 7,032 | 8,352 | 9,378 |
| | No. of genes (>= fold25 & < 1RPKM) | 4 | 130 | 34 | 3 | 402 | 227 | 117 | 42 |
| | No. of genes (>= fold25 & >= 1RPKM) | 8 | 1,476 | 770 | 7 | 3,611 | 7,002 | 2,200 | 7,830 |
| Experimental factors | Cell type | GM12878 | H1-hESC | K562 | HepG2 | HUVEC | MCF-7 | NHEK | DLD-1 |
| --- | --- | --- | --- | --- | --- | --- | --- | --- | --- |
| H3K4me3 | No. of genes | 5,023 | 3,668 | 4,015 | 6,802 | 3,784 | 3,559 | 4,581 | 2,022 |
| Pol2 | No. of genes | 4,824 | 4,141 | 4,529 | 3,175 | 4,554 | 2,625 | 4,148 | 2,894 |
| H3K36me3 | No. of genes | 2,293 | 2,143 | 2,593 | 1,915 | 2,026 | 201 | 2,491 | 1,077 |
| all positive | No. of genes | 1,258 | 880 | 1,742 | 1,233 | 1,197 | 95 | 1,605 | 379 |
| | No. of genes (>= fold10) | 352 | 697 | 1,199 | 63 | 972 | 95 | 1,192 | 341 |
| | No. of genes (< 1RPKM) | 822 | 571 | 1,210 | 824 | 923 | 43 | 1,214 | 55 |
| | No. of genes (>= 1RPKM) | 436 | 309 | 532 | 409 | 274 | 52 | 391 | 324 |
| | No. of genes (>= fold10 & < 1RPKM) | 213 | 423 | 769 | 48 | 734 | 43 | 884 | 50 |
| | No. of genes (>= fold10 & >= 1RPKM) | 139 | 274 | 430 | 15 | 238 | 52 | 308 | 291 |
| | No. of genes (>= fold25 & < 1RPKM) | 7 | 66 | 46 | 0 | 319 | 40 | 194 | 35 |
| | No. of genes (>= fold25 & >= 1RPKM) | 0 | 53 | 49 | 3 | 121 | 41 | 96 | 248 |
Supplementary Figure 11

## Slide 17
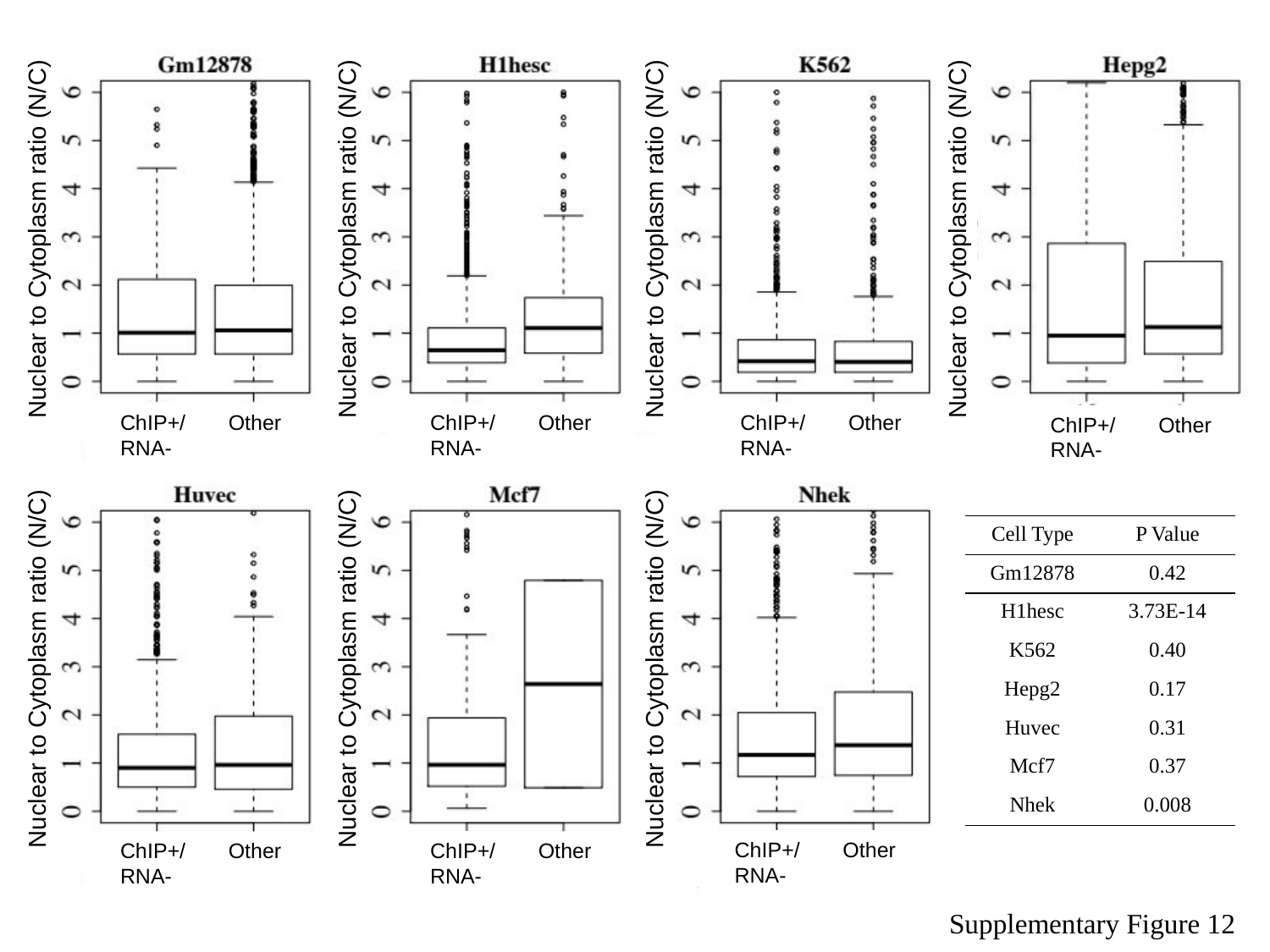

Nuclear to Cytoplasm ratio (N/C)
Nuclear to Cytoplasm ratio (N/C)
Nuclear to Cytoplasm ratio (N/C)
Nuclear to Cytoplasm ratio (N/C)
ChIP+/RNA-
Other
ChIP+/RNA-
Other
ChIP+/RNA-
Other
ChIP+/RNA-
Other
| Cell Type | P Value |
| --- | --- |
| Gm12878 | 0.42 |
| H1hesc | 3.73E-14 |
| K562 | 0.40 |
| Hepg2 | 0.17 |
| Huvec | 0.31 |
| Mcf7 | 0.37 |
| Nhek | 0.008 |
Nuclear to Cytoplasm ratio (N/C)
Nuclear to Cytoplasm ratio (N/C)
Nuclear to Cytoplasm ratio (N/C)
ChIP+/RNA-
Other
ChIP+/RNA-
Other
ChIP+/RNA-
Other
Supplementary Figure 12
